# Callosal responses in a retrosplenial column

**DOI:** 10.1007/s00429-017-1529-5

**Published:** 2017-10-28

**Authors:** Alejandro Sempere-Ferràndez, Belén Andrés-Bayón, Emilio Geijo-Barrientos

**Affiliations:** Instituto de Neurociencias, Universidad Miguel Hernández-Consejo Superior de Investigaciones Científicas, Campus de San Juan, Avenida Ramón y Cajal s/n, 03550 San Juan de Alicante, Spain

**Keywords:** Corpus callosum, Cortical circuits, Interhemispheric communication, Pyramidal neuron, Inhibitory neuron, Synaptic transmission

## Abstract

The axons forming the corpus callosum sustain the interhemispheric communication across homotopic cortical areas. We have studied how neurons throughout the columnar extension of the retrosplenial cortex integrate the contralateral input from callosal projecting neurons in cortical slices. Our results show that pyramidal neurons in layers 2/3 and the large, thick-tufted pyramidal neurons in layer 5B showed larger excitatory callosal responses than layer 5A and layer 5B thin-tufted pyramidal neurons, while layer 6 remained silent to this input. Feed-forward inhibitory currents generated by fast spiking, parvalbumin expressing  interneurons recruited by callosal axons mimicked the response size distribution of excitatory responses across pyramidal subtypes, being larger in those of superficial layers and in the layer 5B thick-tufted pyramidal cells. Overall, the combination of the excitatory and inhibitory currents evoked by callosal input had a strong and opposed effect in different layers of the cortex; while layer 2/3 pyramidal neurons were powerfully inhibited, the thick-tufted but not thin-tufted pyramidal neurons in layer 5 were strongly recruited. We believe that these results will help to understand the functional role of callosal connections in physiology and disease.

**Electronic supplementary material:**

The online version of this article (doi:10.1007/s00429-017-1529-5) contains supplementary material, which is available to authorized users.

## Introduction

The corpus callosum is one of the major fiber bundles of the brain of placental mammals, including humans. Callosal fibers sustain interhemispheric communication between homotopic cortical areas along the entire rostro-caudal axis of the cortex (Yorke and Caviness 1975) and are known to have a prominent role in sensorimotor integration (Hubel and Wiesel 1967; Diedrichsen et al. 2003; Schulte and Müller-Oehring 2010) and high-order cognitive functions (Luck et al. 1989; Gazzaniga 2005; Glickstein and Berlucchi 2008), including self-perception (Uddin 2011).

Despite the early postnatal brain contains some callosal fibers immunoreactive to gabaergic markers (Kimura and Baughman 1997), in the young and mature brain, callosal projecting neurons (CPNs) are mainly, if not entirely, pyramidal neurons (Le Bé et al. 2007; Ramos et al. 2008), and their synapses on contralateral circuits are excitatory (Kumar and Huguenard 2001, 2003). CPNs are not a homogeneous population (Molyneaux et al. 2009; Fame et al. 2016), but can be grouped at least in three categories according to their laminar position (reviewed in Fame et al. 2011); about 80% of them belong to superficial layers, while smaller populations can be found in layers 5 and 6. Contralateral targets of callosal fibers include not only different subtypes of pyramidal cells (Vogt and Gorman 1982; Kawaguchi 1992; Kumar and Huguenard 2001, 2003) but also inhibitory interneurons (Carr and Sesack 1998; Cissé et al. 2003, 2007; Karayannis et al. 2007; Petreanu et al. 2007), which in turn innervate local pyramidal neurons. Therefore, the effect of this cortical input on their postsynaptic targets will depend on the balance between the direct callosal excitation and the disynaptic inhibitory component (Kawaguchi 1992; Chowdhury and Matsunami 2002; Irlbacher et al. 2006).

It is known that response synchronization between neurons of homotopic areas from both cortical hemispheres disappear after callosotomy (Engel et al. 1991), indicating that interhemispheric communication has an integrative function coordinating distal equivalent circuits in a single computational unit (Schmidt et al. 2010). Nonetheless, evidence for a net inhibitory role of the corpus callosum also exists (Hlushchuk and Hari 2006; Reis et al. 2008; Beaulé et al. 2012; Palmer et al. 2012). Accordingly, it has been proposed that callosal axons sustain competition between contralateral ensembles, leading to lateral dominance (for a review on these two opposed hypothesis see Bloom and Hynd 2005; van der Knaap and van der Ham 2011).

The lack of a detailed description of the connectivity between callosal projecting neurons (CPNs) and their contralateral targets remains as a major limitation in our understanding of the functional role of the callosal transfer. Our aim was to fill this gap by studying the influence of the CPNs on contralateral cortical microcircuits. Despite several attempts have been done to characterize the impact of CPNs on contralateral circuits (Karayannis et al. 2007; Palmer et al. 2012; Lee et al. 2014; Rock and Apicella 2015), so far, this is the first study considering the contribution of this pathway within the entire columnar extension of the contralateral cortex. For this, we have performed a detailed electrophysiological screening across different categories of pyramidal and gabaergic neurons in the retrosplenial cortex, a high-order association area involved in spatial cognition and context recognition (Wolbers and Büchel 2005; Smith et al. 2012; Czajkowski et al. 2014; reviewed in Vann et al. 2009).

## Materials and methods

### Ethical approval

Mice were maintained, handled, and sacrificed in accordance with national and international laws and policies (Spanish Directive “Real Decreto 1201/2005”; European Community Council Directive 86/609/EEC). The Ethical Committee for the Experimental Research of the Universidad Miguel Hernández approved the experimental protocols.

### Slice preparation

Brain slices of neocortex were prepared from mice of either sex (C57-BL6 strand; 18–21 postnatal days). Animals were killed by cervical dislocation and their brains were quickly excised and submerged in ice-cold low Ca^2+^/high Mg^2+^ cutting solution (composition in mM: NaCl 124, KCl 2.5, NaHCO_3_ 26, CaCl_2_ 0.5, MgCl_2_ 2, NaH_2_PO_4_ 1.25, glucose 10; pH 7.4 when saturated with 95% O_2_ + 5% CO_2_). Coronal slices (350 μm thick) were cut using a vibratome (Leica VT-1000; Germany) and transferred to a glass beaker, in which the tissue was submerged in artificial cerebrospinal fluid (ACSF; composition in mM: NaCl 124, KCl 2.5, NaHCO_3_ 26, CaCl_2_ 2, MgCl_2_ 1, NaH_2_PO_4_ 1.25, glucose 10; pH 7.4 when saturated with 95% O_2_ + 5% CO_2_) at 34 °C for 30 min. The slices were stored submerged in ACSF for at least 1 h at room temperature before recordings were made. One slice at a time was transferred to a submersion-type recording chamber, and kept at 32–34 °C during the recording period. The ACSF used to bath the slices was fed into the recording chamber at a rate of 2–3 ml min^−1^ and was continuously bubbled with a gas mixture of 95% O_2_ + 5% CO_2_.

### Slice stimulation

**Fig. 1:**
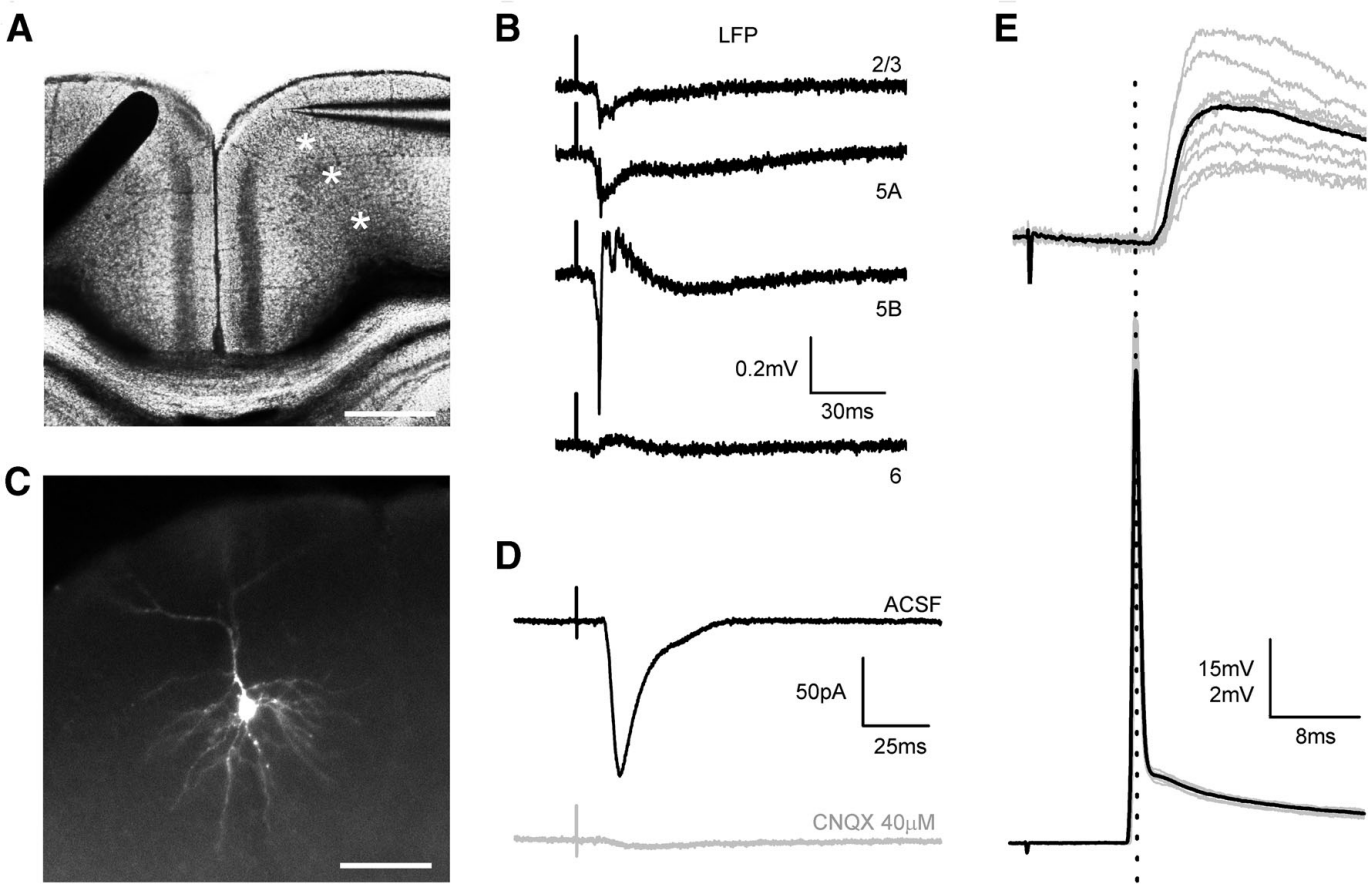
Coronal slices conserving callosal connections. **a** DIC microphotographs of a cortical slice containing the anterior part of the RSC and showing the position of the stimulus electrode (black bar at the left) on the superficial layers of one hemisphere and a recording pipette (right) in the contralateral homotopic region. Scale bar 450 µm. **b** LFP recordings in L2/3, L5A, L5B and L6 (sites marked with asterisks in panel **a**) in response to contralateral stimulation (each trace is the average of ten consecutive responses). **c** Pyramidal neuron from layer 2/3 observed by fluorescence microscopy after intracellular staining with Alexa Fluor 594. Scale bar 50 µm. **d** Postsynaptic current evoked in the pyramidal neuron from C (stimulus intensity 200 µA) with the stimulus configuration shown in **a**. Notice that the application of the AMPA receptor antagonist CNQX (40 μM) blocked the response (gray trace). Each trace is the average of ten responses. **e** PSPs evoked in a superficial pyramidal neuron by a 400 µA stimulus (upper panel). Increasing the stimulus intensity to 500 µA evoked an antidromic spike (lower panel). Gray traces are successive responses and the black trace is the average

Slices were stimulated with a concentric bipolar electrode (CBAFC75 outer diameter 125 µm, Frederick Haer & Co, USA) placed on layer 2/3 of the homotopic contralateral cortex with respect to the recording region. Integrity of the projection was assessed by extracellular recordings prior to intracellular experiments (see Fig. 1a, b). Single pulses (stimulus intensity 100–500 µA, 0.1 ms) were applied at a frequency of 0.2 Hz to recruit CPNs contralateral to the recording site.

### Intracellular recordings

Somatic whole-cell recordings from neurons located all across the cortical depth of the anterior part of agranular RSC cortex (− 2.30 to − 1.70 from Bregma) were made under visual control using an upright microscope (Olympus BX50WI) equipped with Nomarski optics and a water immersion lens (40×). Recordings were obtained in current-clamp and/or voltage-clamp mode with a patch-clamp amplifier (Multiclamp 700B, Molecular Devices, USA). No correction was made for the pipette junction potential (which was estimated to be about − 10 mV using the junction potential calculator included in the pClamp software). Voltage and current signals were filtered at 2–4 kHz and digitized at 20 kHz with a 16-bit resolution analog to digital converter (Digidata 1322A, Axon Instruments). The generation and acquisition of pulses were controlled by pClamp 9.2 software (Axon Instruments). Patch pipettes were made from borosilicate glass (1.5 mm o.d., 0.86 mm i.d., with inner filament) and had a resistance of 4–7 MΩ when filled.

Current-clamp experiments were performed with an intracellular solution containing (in mM): 130 K-gluconate, 5 KCl, 5 NaCl, 5 EGTA, 10 HEPES, 2 Mg-ATP, 0.2 Na-GTP, 0.01 Alexa Fluor 594; pH 7.2 adjusted with KOH; 285–295 mOsm). Voltage-clamp recordings were performed with an intracellular solution containing (in mM): 135 Cs-methane sulfonate, 10 NaCl, 5 EGTA, 10 HEPES, 2 Mg-ATP, 0.2 Na-GTP, 0.01 Alexa Fluor 594; pH 7.2 adjusted with CsOH; 285–295 mOsm. The theoretic Nernst equilibrium potentials for the K-based internal solution were (in mV): *E*_K_ = − 105.7, *E*_Na_ = 89.3, *E*_Cl_ = − 68.5). The theoretic Nernst equilibrium potentials for the Cs-based internal solution were (in mV): *E*_Na_ = 71.4, *E*_Cl_ = − 68.5.

Current clamp recordings were performed at the resting membrane potential of the neuron. Series resistance (*R*_s_) was measured and balanced on-line under visual inspection assisted by the Bridge Balance tool of Clampex software. *R*_s_ was monitored at the beginning and at the end of each protocol, and re-balanced if needed. Cells in which *R*_s_ > 40 MΩ were discarded (typical *R*_s_ < 25 MΩ).

For voltage clamp experiments, EPSCs and IPSCs were recorded with holding potentials of − 70 and 0 mV, the measured reversal potential for the inhibitory and excitatory synaptic currents, respectively. Neurons in which *R*_s_ > 30 MΩ were discarded (typical *R*_s_ was between 10 and 25 MΩ). The error in the measure of the membrane potential (*V*_e_) was computed as *V*_e_ = *I*_hold_ × *R*_s_; *I*_hold_ stands for the holding current needed to set the holding potential (*V*_h_). To hold the cell at the desired membrane potential (*V*_m_), we set *V*_h_ as *V*_h_ = *V*_m_ + *V*_e_. Quantification of intrinsic membrane properties and synaptic responses was performed on Clampfit10.3.

### Neuron-type identification

Each neuron recorded was assigned to a cortical layer according to the position of the soma (distance to pia was measured for each cell). Recorded cells were identified as pyramidal neurons by their intracellular perfusion with the fluorescent dye Alexa Fluor 594, which was added to the recording solution. These neurons showed a characteristic pyramidal soma and a dominant apical spiny dendrite oriented to layer I while basal dendritic arbors were tangentially oriented (see example in Figs. 1c, 3f, 7a).

**Fig. 2:**
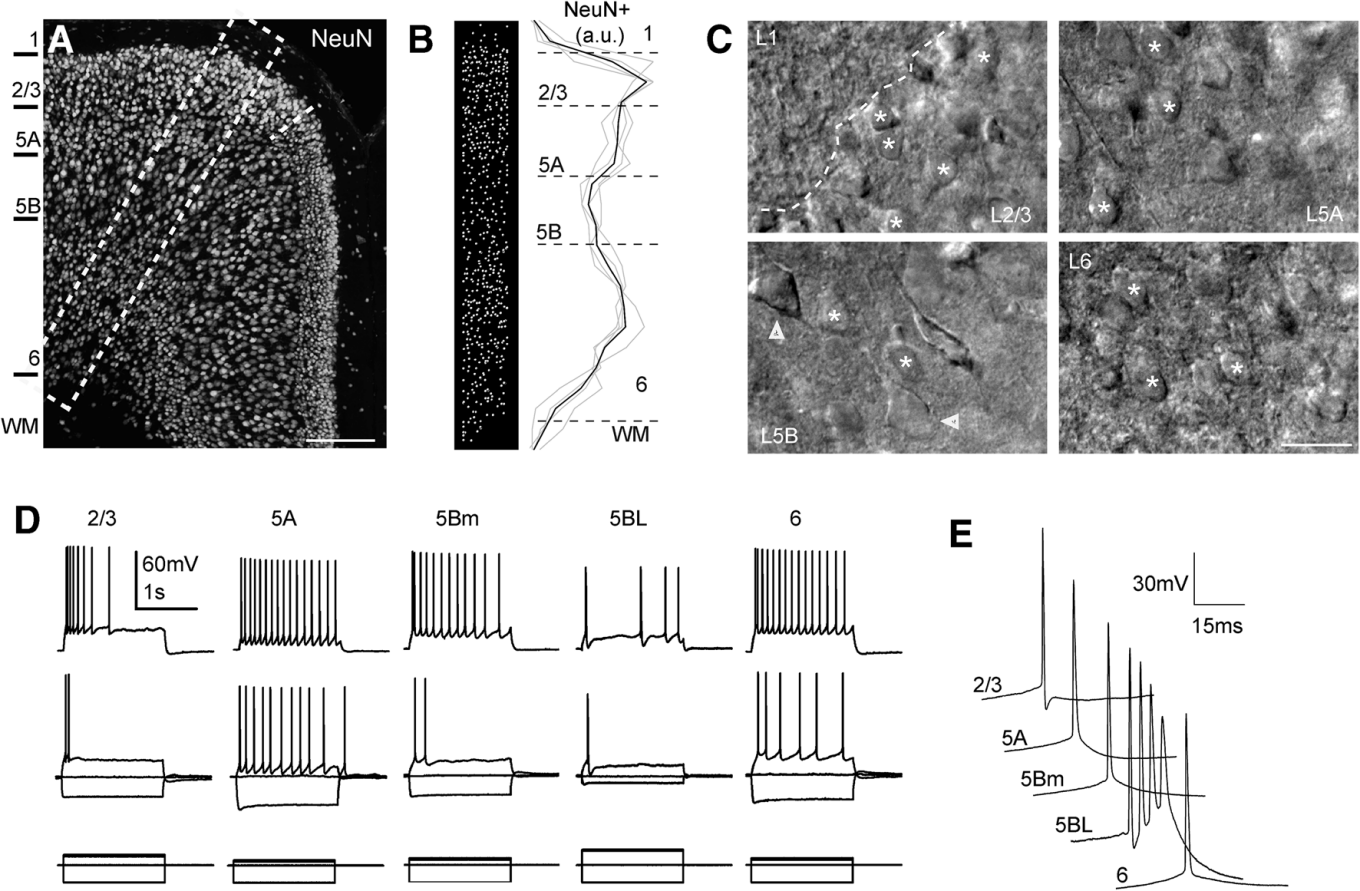
Pyramidal neurons in the agranular RSC. **a** Confocal z-stack image of the anterior RSC immunostained for the neuronal marker NeuN. The short discontinuous line indicates the limit between the agranular RSC (aRSC, dorsal) and the granular RSC (gRSC, ventral). **b** Left, Imaris reconstruction of the position of NeuN positive somas from neurons in the columnar inset shown in A (546 NeuN positive cells); right, NeuN positive cell density across an aRSC column measured with a 50 µm bin width (*n* = 7 slices from four mice; selected columns were 300–400 µm wide, mean number of NeuN positive cells per column: 466). Grey traces represent individual cases and the black trace the mean. **c** DIC microphotographs of the agranular RSC in a slice placed in a recording chamber showing the sizes of the bodies of the neurons found in each layer. Note the presence of neurons with medium–small sizes (marked with asterisks) in all layers and the presence of two L5BL neurons (soma marked with arrowheads) in layer 5B whose soma was clearly larger. **d** Membrane voltage responses from a L2/3, L5A, L5Bm, L5BL and a L6 pyramidal neuron to 1500 ms current pulses. Responses were recorded at resting membrane potential (*I*_holding_ = 0 pA). **e** First action potential from the first suprathreshold response shown at larger scales. Scale bars 250 and 20 µm in **a** and **c**, respectively

Neurons were classified according to the laminar localization of their somas. An important hallmark of the laminar organization of the agranular retrosplenial cortex (and of other cortical areas) is the presence of a population of pyramidal neurons with large somas in layer 5B; these neurons were easily identified in slices with DIC optics and a 40× lens (see Fig. 2c, lower left panel). Taking into account the presence of large neurons in layer 5B, the assignation of neurons to different layers was as follows (see also the results section): layer 2/3 neurons (somas up to 300 μm from the pia); layer 5A neurons (somas between 300 μm and the level of large pyramidal neurons); layer 5B neurons (somas in the layer with large pyramidal neurons; these large neurons were between 410 and 620 μm from the pia) and layer 6 (somas below the level of large pyramidal neurons). To assess the presence of these larger neurons in layer 5B we measured the somas of neurons across layers 2/3–6 in snapshots taken with DIC optics and the 40× lens (Online Resource Fig. 1; Table 1); this quantification revealed the presence of neurons with large somas (> 200 μm^2^), which characterizes layer 5B. The pyramidal neurons of layer 2/3, 5A and 6 were of medium size (somatic area < 200 μm^2^ as seen under DIC optics) and quite homogeneous. In contrast, in layer 5B it was clear the presence of two populations of pyramidal neurons: medium-sized pyramidal neurons similar to those of layers 2/3, 5A and 6 and the large pyramidal neurons, which had very different morphological and electrophysiological properties than their neighboring medium-sized neurons (see “Results”).

**Table 1:** Passive electrophysiological properties of pyramidal and gabaergic interneurons in the agranular RSC

	*n*	Age (days)	Distance to pia (µm)	*V*_rest_ (mV)	*R*_m_ (peak) (MΩ)	*V*_sag_	*τ*_m_ (ms)
Pyr 2/3	23	19.2 ± 1.1	190.6 ± 33.0	− 75.5 ± 4.1	166.4 ± 55.0	0.04 ± 0.03	22.6 ± 7.7
Pyr 5m	25	19.9 ± 1.4	396.0 ± 71.5	− 65.2 ± 5.4	211.7 ± 67.6	0.11 ± 0.06	21 ± 4.9
Pyr 5BL	21	19.4 ± 0.9	547.2 ± 71.1	− 65.7 ± 2.2	46.9 ± 13.2	0.25 ± 0.04	11.8 ± 2.0
Pyr 6	17	19.5 ± 0.8	941.6 ± 114.5	− 72.7 ± 5.2	215.0 ± 62.7	0.16 ± 0.05	15.9 ± 4.1
PV-FS	45	19.7 ± 1.2	452.7 ± 217.7	− 72.1 ± 5.1	70.8 ± 24.0	0.10 ± 0.04	5.9 ± 1.9
Non PV-FS	16	19.2 ± 1.3	359.7 ± 206.5	− 73.0 ± 2.8	196.7 ± 81.5		9.2 ± 5.4

Resting membrane potential (*V*_rest_) was measured as the average potential of a 5 s time window after going into whole cell configuration; membrane input resistance (*R*_m_) was computed from the slope of the linear fit from steady state voltage responses to low amplitude hyperpolarizing current square steps; membrane time constant (*τ*_m_) was estimated form the voltage response to a hyperpolarizing current step < 50 pA; voltage sag ratio (*V*_sag_) was calculated from the voltage response to a – 300 pA square current injection of 1 s as the ratio between the steady state and peak membrane potential. The Pyr 5m group includes 5A and 5Bm pyramidal neurons. Data are shown as mean ± SD

Parvalbuming-expressing fast spiking interneurons (PV-FS) were identified by their characteristic spiking pattern, briefly, tonic discharges of narrow action potentials with little accommodation and large after-hyperpolarization in response to suprathreshold square current pulses. To increase the probability of patching PV-FS interneurons, we used the *Pvalb*-*Cre;RCE* mouse in which PV-expressing neurons are labeled with GFP; these animals were made by crossing *Palvb*-*Cre* mice (Hippenmeyer et al. 2005) with a Cre-dependent EGFP reporter line RCE;FRT (Sousa et al. 2009); or the GAD67-GFP line (Tamamaki et al. 2003), in which all types of gabaergic interneurons are labeled with GFP. Non PV-FS neurons were identified as GFP positive neurons from the GAD67-GFP line lacking the typical electrophysiological properties of FS cells.

For morphological reconstruction of recorded neurons with biocytin we used the method described by Marx et al. (2012). Briefly, biocytin was added to the intracellular solution at a final concentration of 5 mg/ml. After recordings, slices were fixed overnight at 4 °C in 100 mM phosphate-buffered saline (PBS; pH 7.4) that contained 4% paraformaldehyde. After several rinses in PBS that contained 1% Triton X-100, the endogenous peroxidase was blocked by incubation in 3% H_2_O_2_. The slices were transferred to a complex of 1% avidin-biotinylated HRP that contained 0.5% Triton X-100 (ABC Peroxidase Standard PK-400 Vectastain; Vector Labs, Burlingame, CA, USA) and were left for 1 h with gentle shaking. The slices were reacted using 3,3-diaminobenzidine (DAB; Sigma) and the reaction was stopped by adding H_2_O_2_ 0.9%. The slices were mounted on glass slides, embedded in Eukitt, and coverslipped. Biocytin-stained neurons were drawn using Neurolucida software (MBF Bioscience, Vermont USA).

### Callosal axon labeling by in utero electroporation

We utilized a green fluorescent protein (GFP) plasmid (pCX-GFP) to label superficial CPNs by in utero electroporation. Plasmid DNA was purified using an extraction midi kit (NucleoBond^®^ xtra midi, Macherey–Nagel). For in utero electroporation, DNA was dissolved at a final concentration of 1 µg/µl in milliqH_2_O with 1% fast green. E16.5 pregnant dams (C57-BL6 strand) were anesthetized with isoflurane. The uterus was accessed via a 1.5 cm incision of the abdominal wall, and individual embryos were injected through the intact uterine wall using glass micro capillaries under a fiber optic light source. After electrodes were placed strategically, 5 pulses of 40 V current of 50 ms duration were applied at intervals of 950 ms using an electroporator (Square Wave model CVY21SC, Nepa Gene). The surgical incision was sutured and mice were administered a single subcutaneous injection of 0.1 mg/kg buprenorphine analgesic (Buprex^®^, Schering Plough), and then orally with 0.03 mg of buprenorphine per food pellet. Electroporated mice at 3–4 postnatal weeks were anesthetized with isoflurane and perfused with PFA 4% in PBS. Brains were removed and post fixed overnight in PFA 4% at 4 °C. 40 µm coronal sections were cut in the vibratome from brains embedded in agarose. Immunohistochemistry to GFP was performed in floating slices with GFP chicken polyclonal antibody 1:500 (GFP1020, AVES) and goat antibody to chicken AlexaFluor488 1:500 (A 11039 Molecular Probes).

### Channelrhodopsin 2 photostimulation of PV-FS neurons

For these experiments, we used slices prepared from animals that expressed Channelrhodopsin 2 in PV-FS interneurons. These animals were obtained by crossing *Pvalb* ^*tm1(cre)Arbr*^ mice with Ai32(RCL-ChR2(H134R)/EYFP) mice. In cortical slices from these animals the PV expressing neurons (which express EYFP and Channelrhodopsin 2) were identified under fluorescence epi illumination. To depolarize these neurons up to the action potential firing we used pulses of blue light (470 nm) generated by a LED light source (SOLIS 1C-from Thorlabs, New Jersey, USA) and controlled by a high-power LED driver (DC2200 from Thorlabs, New Jersey, USA). The blue light was applied to the slice through the epi-illumination system of the microscope and the 40× water immersion objective, which resulted in the stimulation of the whole view of field of the objective. We used pulses of ~ 55% of the maximum power of the light source.

### Statistics

Data are shown as the mean ± standard error of the mean (SEM), and the number of cases, unless otherwise indicated. For comparison of the distribution of one variable among two non-paired samples, the two-tailed Mann–Whitney rank sum test was employed. In the case of neuron pairs recorded either simultaneously or sequentially (for the latter case, the position of the stimulus electrode and the intensity was kept constant), the two-tailed Wilcoxon signed rank test was employed. For comparison of proportions among two samples, the two-tailed *Z* score test for two population proportions was employed. For linear correlation among two variables, the Pearson correlation coefficient was computed. For analysis of variance across more than two samples, the one-way ANOVA test was employed. If significant (*p* value < 0.05), Bonferroni post hoc corrections were applied to compare across all two sample possibilities. Statistical analysis were performed on OriginPro8 (Origin Lab Corporation) and Sigma Stat 3.11 (Systat Software Inc.). The degree of statistical significance is indicated by * (*p* < 0.05), ** (*p* < 0.01) or *** (*p* < 0.001).

## Results

Coronal slices including the anterior region of the RSC maintained the integrity of the callosal pathway across hemispheres (Fig. 1). Local field potentials and intracellular recordings indicated that electrical stimulation with a bipolar electrode placed on the superficial layers of the agranular RSC evoked synaptic responses in the homotopic contralateral cortex (Fig. 1) that were blocked by the application of the AMPA receptor antagonist CNQX 40 µM (Fig. 1d); this allowed us to study the physiology and connectivity of callosal synapses in cortical circuits.

**Fig. 3:**
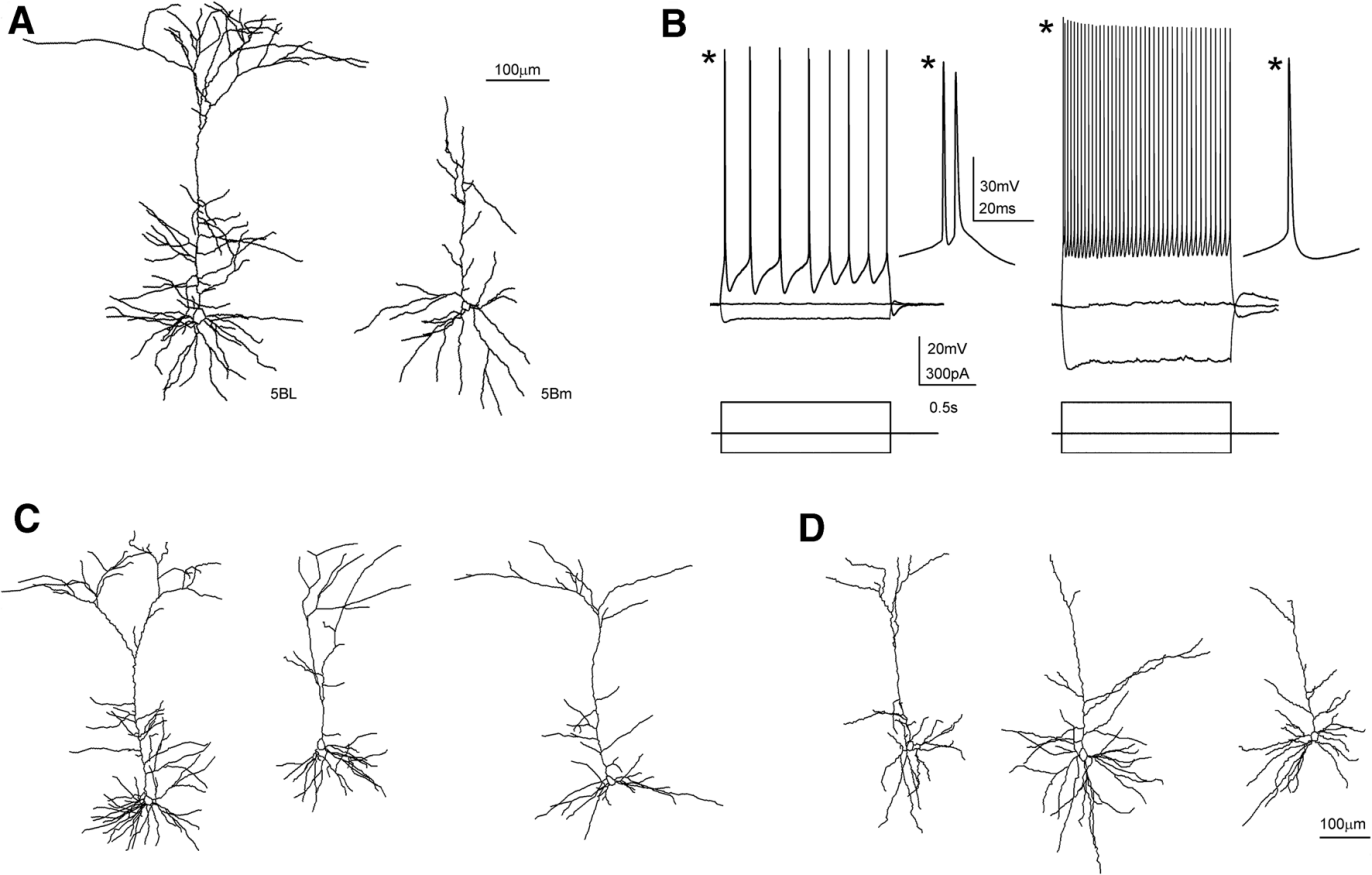
Morphological and electrophysiological properties of L5BL and L5Bm pyramidal neurons of the retrosplenial cortex. **a** Example of a large (L5BL; left) and a medium-sized soma (L5Bm; right) pyramidal neuron from layer 5B that were labelled with biocytin and morphologically reconstructed. Notice the presence of a thick apical tuft in the L5BL but not in the L5Bm pyramidal neuron. **b** Responses to square current steps in the same neurons as in **a**. Notice the presence of bursts of APs in response to the suprathreshold current pulse and the lower input resistance of the L5BL when compared to the L5Bm pyramidal neuron. **c** Example of three more L5BL pyramidal morphologically reconstructed after biocytin labelling. **d** Same as in **d** for L5Bm pyramidal cells. Notice the absence or small size of the apical tuft in L5Bm when compared to L5BL pyramidal neurons

However, given the symmetry of the callosal projection, electrical stimuli applied to layer 2/3 could also stimulate antidromically contralateral neurons projecting to the stimulus site, therefore making possible that the evoked postsynaptic responses were a mixture of contralateral (callosal) and ipsilateral inputs. To assess the contribution of this non-desired source of synaptic input, we quantified the ratio of contralateral pyramidal neurons in which antidromic spikes were evoked (Fig. 1e). Antidromic spikes were easily identified as low latency jitter, all-or-none action potentials arising directly from the resting membrane potential (see an example in Fig. 1e and compare with ortodromic spikes in Fig. 3d). In our sample of layer 2/3 neurons, where most CPNs are located (Fame et al. 2011), antidromic responses were quite uncommon: with stimulus intensities of 100 μA we observed antidromic spikes in 1 out of 92 neurons; with 200 µA in 2/90 neurons, and with 500 µA in 6/76 neurons. In the pyramidal neurons recorded in deeper layers we never recorded an antidromic spike. This very low proportion of neurons showing antidromic spikes indicated that in our experimental conditions, and at least when using stimuli of low and medium strength (100–200 µA), we stimulated mainly the soma of ipsilateral CPN neurons and not the axons of contralateral neurons and therefore the observed synaptic responses were mostly induced by callosal input, being minimal the contribution of local synapses activated antidromically.

### Pyramidal neuron diversity in the agranular RSC

We divided the agranular retrosplenial cortex (RSC) in 5 layers (1, 2/3, 5A, 5B and 6) according to changes in neuronal density measured by neuron soma counts in NeuN stained slices (Fig. 2a, b). This laminar distribution is in agreement with previous cytoarchitectonic studies on the mouse RSC, including the fact that in the mouse anterior agranular RSC, layer 4 is almost undetectable (Vogt and Paxinos 2014). Recorded neurons were assigned to one of this layers according to the criteria described in methods. Medium-size regular spiking neurons with spiny vertically oriented apical dendrites (see one example in Fig. 1c) were recorded all through layers 2–6. These neurons responded with adapting trains of action potentials in response to suprathreshold current pulses (Fig. 2d) and were considered as pyramidal neurons.

Layer 5B contained both medium and large pyramidal neurons (see methods). During the recording sessions in layer 5B we recorded from medium sized pyramidal neurons (L5Bm) but we also selected pyramidal neurons whose soma was clearly larger than the soma of other neighboring pyramidal neurons, and also larger than the soma of neurons in layers 2/3, 5A or 6 (see Fig. 2c, lower left panel). We measured the somatic area (from the snapshots taken under DIC optics) of a subset of neurons that we selected and recorded as large layer 5B pyramidal neurons (*n* = 19); their somas had an area of 306.2 ± 57.3 μm^2^ (range 210.0–414.3 μm^2^). These values were always above 200 μm^2^ and corresponded to the large neurons that we identified in layer 5B (see Online Resource Fig. 1). It is important to note that all neurons that we identified and recorded in layer 5B as large pyramidal neurons had always an input resistance < 80 MΩ (43.9 ± 12.3 MΩ; range 19.7–72.3 MΩ; *n* = 51). In contrast, those neurons identified and recorded as medium-size pyramidal neurons had input resistance > 130 MΩ (198.9 ± 53.2 MW; range 133.0–300.1 MΩ; *n* = 15). We classified these large neurons as L5BL and the medium sized neurons as L5Bm and, despite our subjective selection based on the somatic size, the fact that the input resistance did not overlap in L5BL and L5Bm pyramidal neurons strongly suggest that we were correctly segregating layer 5B neurons in two different subtypes. To further characterize both types of neurons in layer 5B we made a detailed “a posteriori” analysis over the whole set of neurons recorded under current-clamp conditions in layer 5B (Online Resource Table 2) and classified as L5BL and L5Bm. This analysis showed that L5BL and L5Bm neurons were clearly different. L5BL and L5Bm neurons had very different passive and active electrical properties (Online Resource Table 2; see also Figs. 2d, e, 3b). L5BL neurons showed a lower membrane input resistance with non-overlapping ranges (as stated above) and a larger voltage sag in the responses to hyperpolarizing current pulses (0.26 ± 0.04 vs 0.16 ± 0.04, *p* < 0.001). Importantly, L5BL neurons also showed a higher probability of firing bursts of action potentials in response to just-threshold current pulses (23 out of 51 neurons vs 0 out of 15; *p* < 0.01; a response was considered a burst when the two initial spikes evoked by a just suprathreshold current pulse had an instantaneous frequency > 150 Hz as shown in Figs. 2d, e, 3d). Finally, to confirm the separation between L5BL and L5Bm neurons, we analyzed the dendritic structure and the somatic size of a subset of layer 5B neurons filled with byocitin (Fig. 3). In those neurons in which the apical dendrite was fully reconstructed (*n* = 4 L5BL and *n* = 4 L5Bm) we observed clear differences in the structures of the apical dendrites. L5BL neurons (Fig. 3a, c) had a large apical tuft that branched extensively in layer 1, while L5Bm neurons (Fig. 3a, c) lacked that apical tuft or it was much smaller. In addition, the area of the soma (measured with the Neurolucida software in byocitin stained preparations) was significantly larger in L5BL than in L5Bm neurons (271.7 ± 15.8 μm^2^, *n* = 6 vs 180.2 ± 26.5 μm^2^ *n* = 5; *p* = 0.017). In the L5BL neurons stained with byocitin the presence of a large apical tuft was correlated with a low input resistance and with a tendency to fire bursts of action potentials. Our electrophysiological and morphological analysis show that our selection of pyramidal neurons based in the somatic size, though is in part subjective, effectively separates two subclasses of neurons in layer 5B. The above data suggest that the neurons that we classified as L5BL correspond to the thick-tufted layer 5B pyramidal neurons with extratelencephalic projections, while those neurons classified as L5Bm correspond to thin-tufted layer 5B pyramidal neurons with cortico-cortical and/or cortico-striatal projections (Molnár and Cheung 2006).

Despite the similarities among the medium-sized regular spiking pyramids recorded from different layers, those from layer 2/3 were more hyperpolarized at rest and had a smaller voltage sag than those in layer 5 (see Table 1). These differences were statistically significant (*p* < 0.05 in both cases) and this fact allowed us to set the limit between layer 2/3 and 5A at 300 μm from the pia. L5Bm pyramidal neurons had similar properties to those of L5A (they are grouped together in Tables 1, 2 as L5 m pyramidal neurons), while in L6 they were more hyperpolarized at rest (as superficial ones), but had a larger voltage sag in response to – 300 pA current steps.

**Table 2:** Action potential and firing properties of pyramidal and gabaergic interneurons in the agranular RSC

	AP_thr_ (mV)	*V*_m_–AP_thr_ (mV)	AP_amp_ (mV)	AP 1/2 width (ms)	Firing frequency (Hz)	Adaptation index
Pyr 2/3	− 40.2 ± 3.0	− 35.2 ± 4.0	93.6 ± 4.7	0.62 ± 0.11	50.6 ± 14.7	2.8 ± 0.9
Pyr 5m	− 40.6 ± 2.9	− 24.6 ± 5.8	92.7 ± 4.7	0.59 ± 0.07	55.1 ± 15.4	2.8 ± 1.0
Pyr 5BL	− 43.1 ± 3.4	− 22.6 ± 3.3	97.1 ± 7.6	0.5 ± 0.07	34.9 ± 9.1	1.1 ± 0.3
Pyr 6	− 37.7 ± 4.1	− 35.0 ± 4.2	85.1 ± 4.5	0.63 ± 0.11	48.0 ± 8.9	1.9 ± 0.7
PV-FS	− 37 ± 4.4	− 35.1 ± 6.2	70.7 ± 7.8	0.21 ± 0.03	171.8 ± 8.9	1.0 ± 0.2
Non PV-FS	− 35.6 ± 5.2	− 37.0 ± 5.7	77.3 ± 8.31	0.47 ± 0.13	109.9 ± 45.7	2.4 ± 0.9

Same neurons as in Table 1. Action potential properties were measured for the first action potential elicited in the first suprathreshold square current step. AP threshold (AP_thr_) was manually estimated, AP potential amplitude (AP_amp_) was measured as the potential difference between AP_thr_ and AP peak, AP width was measured at half-amplitude (AP 1/2 width). Firing frequency and adaptation index were obtained for the responses to 575 pA current pulses. Adaptation index was computed as the ratio between 2nd and 3rd AP time interval/last AP time interval. Data are shown as mean ± SD

In summary, we grouped the pyramidal neurons in five categories: L2/3, L5A, L5B medium-size (L5Bm), L5B large-size (L5BL) and L6 pyramidal neurons. A summary of the intrinsic properties of these neurons is given in Tables 1 and 2. Overall, this is the general scheme of pyramidal organization across layers described in other neocortical regions (Connors and Gutnick 1990).

### Larger callosal responses in L2/3 and L5BL pyramidal neurons

**Fig. 4:**
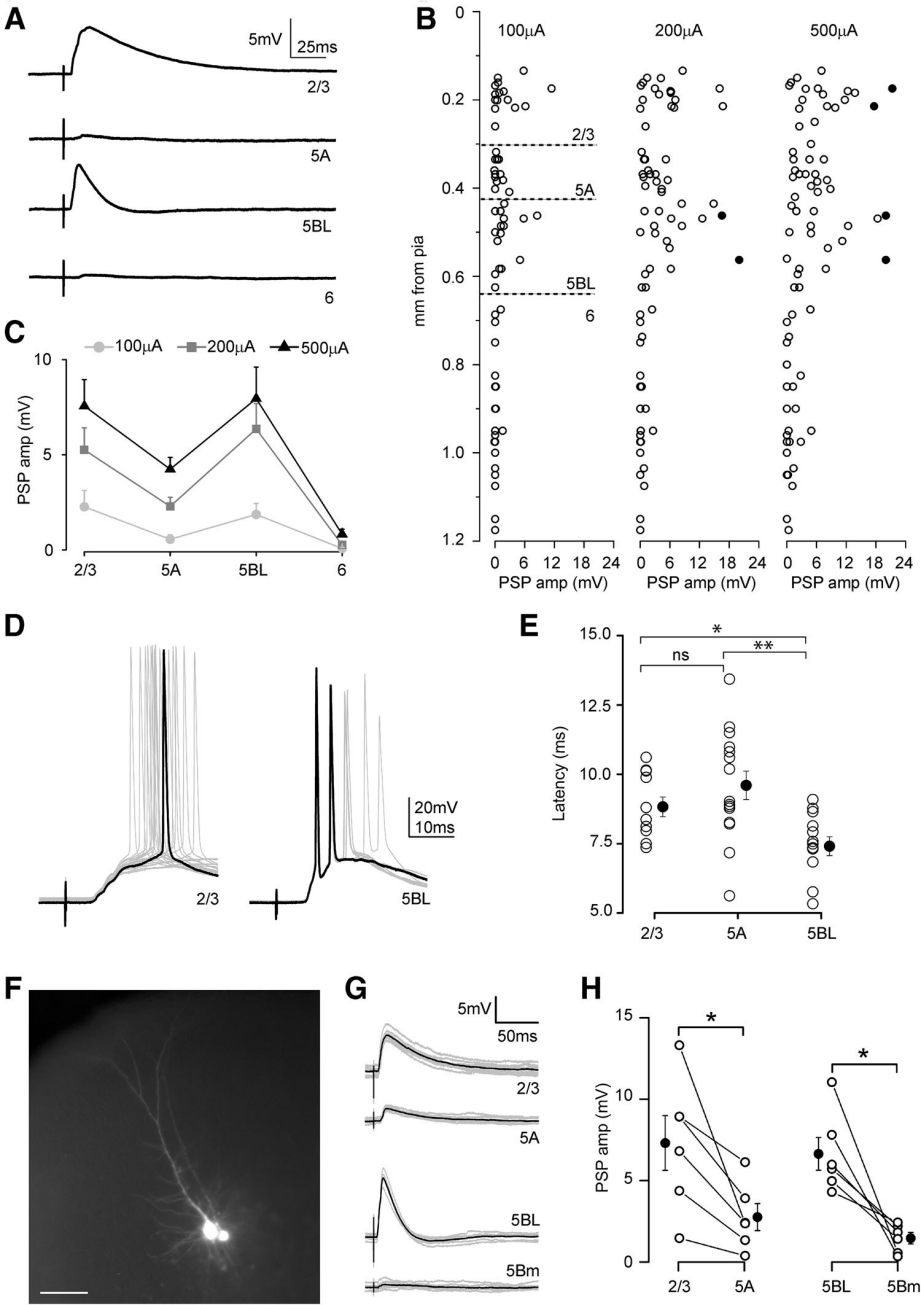
Laminar organization of callosal connections in the agranular RSC. **a** An example of callosal PSPs recorded sequentially from four pyramidal neurons from the same cortical column in response to single pulse stimulation of the contralateral cortex (stimulus intensity 200 µA). Each trace is the mean of at least ten responses. **b** Callosal PSP peak amplitude in a sample of pyramidal neurons (*n* = 21 L2/3, 17 L5A, 19 L5BL and 23 L6 pyramidal neurons, *n* = 16 slices from 16 mice) at three stimulus intensities (100, 200 and 500 µA). Each circle is an individual neuron and filled circles represent neurons in which the firing of action potentials was elicited. Soma to pia distance in µm was (mean ± SD): L2/3 = 190 ± 31, L5A = 371 ± 39, L5BL = 526 ± 71, L6 = 927 ± 136. **c** Mean ± SEM of the PSP peak amplitude across pyramidal categories from the sample shown in **b**. **d** Example of two neurons in which firing was evoked by suprathreshold postsynaptic potentials (stimulus intensity 500 µA). Each trace is an individual response and one of the responses is highlighted in black. **e** Onset latency (measured from stimulus artifact onset to 10% of PSP peak amplitude) of the callosal PSPs evoked in L2/3, L5A and L5BL pyramids. Only slices in which at least 1 neuron of each category were sequentially recorded were analyzed (*n* = 14 L2/3, 15 L5A and 11 L5BL pyramidal neurons; 8 slices of 8 mice; stimulus intensity 200 µA, L6 neurons were excluded). **f** Fluorescence microphotograph of a L5BL (left) vs L5Bm (right) pair of pyramidal neurons simultaneously recorded. **g** Example of the responses evoked in a L2/3 vs 5A pair and in a L5BL vs L5Bm pair of pyramidal neurons simultaneously recorded (stimulus intensity 200 µA). **h** Callosal PSP peak amplitude in a sample of L2/3 vs L5A pairs (left panel, *n* = 6) and L5BL vs L5B-m pairs (right panel, *n* = 6). Stimulus intensity 200 µA. Grey traces show individual responses and the black trace is the average. PSPs were recorded at resting membrane potential

The post-synaptic potentials (PSPs) recorded in L2/3 (*n* = 21), L5A (*n* = 17), L5BL (*n* = 19) and L6 (*n* = 23) pyramidal neurons in response to callosal input are shown in Fig. 4. At all three stimulus intensities tested (100, 200 and 500 µA) the amplitude of the callosal PSPs exhibited a bimodal distribution, being larger in L2/3 and L5BL than in L5A and L6 pyramidal neurons (Fig. 4b, c; see an example in Fig. 4a). A one-way ANOVA analysis of the data obtained at 200 µA stimulus revealed significant differences among the groups (*F* = 10.44, *p* < 0.00001). A Bonferroni post hoc analysis only found significant differences in the amplitude of the response between L2/3, L5A and L5BL with L6 pyramidal cells (Bonferroni corrected *p* value 0.007, computed p-value for Mann–Whitney two-sample comparisons: L2/3 vs L5A 0.080, L2/3 vs L5BL 0.749, L2/3 vs L6 2.6 × 10^−6^, L5A vs L5BL 0.022, L5A vs L6 5.4 × 10^−6^, L5BL vs L6 5.6 × 10^−7^). Within this sample, only in 2/21 L2/3 and 2/19 L5BL pyramidal neurons the callosal PSP were able to fire action potentials (see examples in Fig. 4d). The two L2/3 pyramidal neurons fired in response to 500 µA stimulus, while the L5BL pyramidal neurons did it with lower stimulus intensity (200 μA). The fact that the bimodal distribution of PSP amplitudes was clearly present with 100 and 200 µA stimulus intensities, when ortodromic and antidromic spikes were rare (or totally absent), strongly suggests that this pattern is mostly established by direct callosal input. The onset latency of the evoked responses is shown in Fig. 4e; for latency measurements we only analyzed neurons from experiments in which at least one pyramidal cell were recorded from layers 2/3, 5A and 5B in the same slice. A one-way ANOVA analysis of these data revealed significant differences in the onset latency across neuron types (*F* = 6.002, *p* value 0.00543). A Bonferroni post hoc analysis found significative differences in the onset latency of the responses of L2/3 and L5A with respect L5BL pyramidal neurons (Bonferroni corrected p-value 0.0167, computed *p* value for two-sample Mann–Whitney comparisons: L2/3 vs L5A 0.169, L2/3 vs L5BL 0.0167, L5A vs L5BL 0.004).

The above data revealed a tendency in the amplitude of the PSPs evoked in L5A pyramidal neurons to be smaller than in L2/3 and L5BL ones. To increase the potency of the statistical analysis, as well as to extend our analysis to L5Bm pyramidal neurons, we performed a set of experiments in which we simultaneously recorded from pairs of L2/3 vs L5A and L5BL vs L5Bm pyramidal cells (Fig. 4g, h, *n* = 6 simultaneous pairs of each type; layer 6 pyramidal cells were excluded given their low responsiveness). In these paired recordings, special care was taken to select neurons whose apical dendrites were radially aligned in the case of L2/3–L5A pairs, and whose somas were closely placed in the case of L5BL–L5Bm pairs (intersomatic distance < 50 µm, see example in Fig. 4f). These experiments revealed that callosal PSPs were significantly larger in L2/3 than in L5A neurons and in L5BL than in L5Bm pyramidal neurons (Fig. 4h), extending our previous results. L5Bm neurons innervate L5BL pyramidal neurons (projection of telencephalic on extratelencephalic projecting neurons in layer 5; Harris and Shepherd 2015); this could cause that callosal responses recorded in L5BL neurons could be larger in part due to the firing of L5Bm neurons. We can discard this possibility because in our experiments none of the recorded L5Bm neurons (from a total sample of 27 neurons recorded in different sets of experiments) fired action potentials in response to the contralateral stimulation. This was due to the very small synaptic responses evoked by these stimuli, which were always smaller than 5 mV or 60 pA (see Fig. 4h, right panel) and to the fact that none of these neurons fired antidromically in response to stimuli applied to contralateral layer 2/3.

**Fig. 5:**
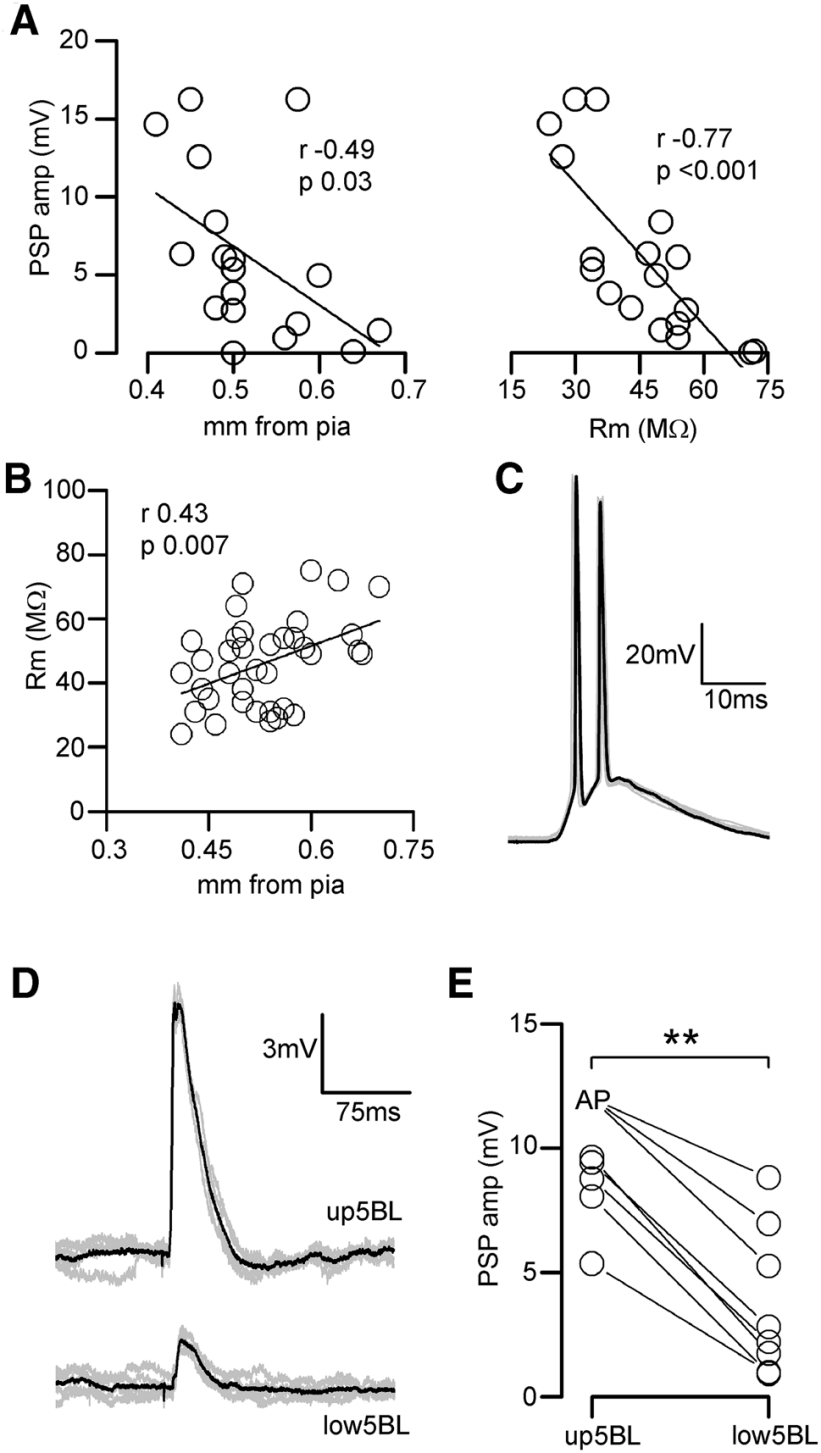
Strong recruitment of upper L5BL pyramidal neurons by callosal input. **a** Callosal PSP peak amplitudes in L5BL pyramidal neurons were negatively correlated with somatic distance from pia (left panel) and with membrane input resistance (right panel); same sample as in Fig. 3a–c; stimulus intensity 200 µA). **b** In L5BL pyramidal neurons, membrane input resistance was positively correlated with somatic distance from pia (*n* = 37). **c** Response of a L5BL pyramidal neuron from the upper half of layer 5B to a 200 µA stimulus. Individual responses are shown in grey and one is highlighted in black. **d** PSPs evoked in an upper vs lower L5BL pyramidal neuron pair evoked by contralateral stimulation. Grey traces are individual responses and the black trace is the average. **e** PSP peak amplitudes in a sample of upper vs lower L5BL pairs (*n* = 8 pairs sequentially recorded in current-clamp; 5 slices from 5 mice; upper L5BL 490 ± 47 µm from pia, lower L5BL 593 ± 61 µm). Stimulus intensity 200 µA. For statistical comparison of PSP amplitudes, in those cells in which firing was evoked we used the PSP amplitude of the largest subthreshold response in the sample

In our sample of L5BL neurons (Fig. 4a–c), callosal PSPs amplitude was negatively correlated with their somatic distance from pia and with their membrane input resistance (Fig. 5a). Moreover, in a wider sample of L5BL pyramidal neurons, we observed that the membrane input resistance of L5BL pyramidal neurons was positively correlated with their columnar depth (Fig. 5b), suggesting the existence of a further specialization between L5BL neurons placed in the upper and the lower part of layer 5B. To test if there was a bias in the callosal connectivity towards upper L5BL neurons, as suggested in Fig. 5a left panel, we compared the callosal PSP amplitude in sequentially recorded pairs formed by of one upper L5BL (whose soma was in the upper half of layer 5B) and one lower 5BL (whose soma was in the lower half of layer 5B) pyramidal neurons. In all cases the callosal PSP was larger in the upper 5BL neuron (Fig. 5d, e). To estimate the firing probability of the upper 5BL we recorded from a total of 14 neurons whose soma was in the upper half of layer 5 (including the 8 neurons from the pairs); in this sample 4/14 neurons (28%) fired by the PSPs evoked by 200 μA contralateral stimuli (see an example in Fig. 5e). These data indicated that, in the agranular RSC, one hemisphere could recruit the extratelencephalic output pathway of the opposed homotopic region by means of the callosal projection.

### Laminar disposition of callosal axon terminal branches from superficial CPNs

**Fig. 6:**
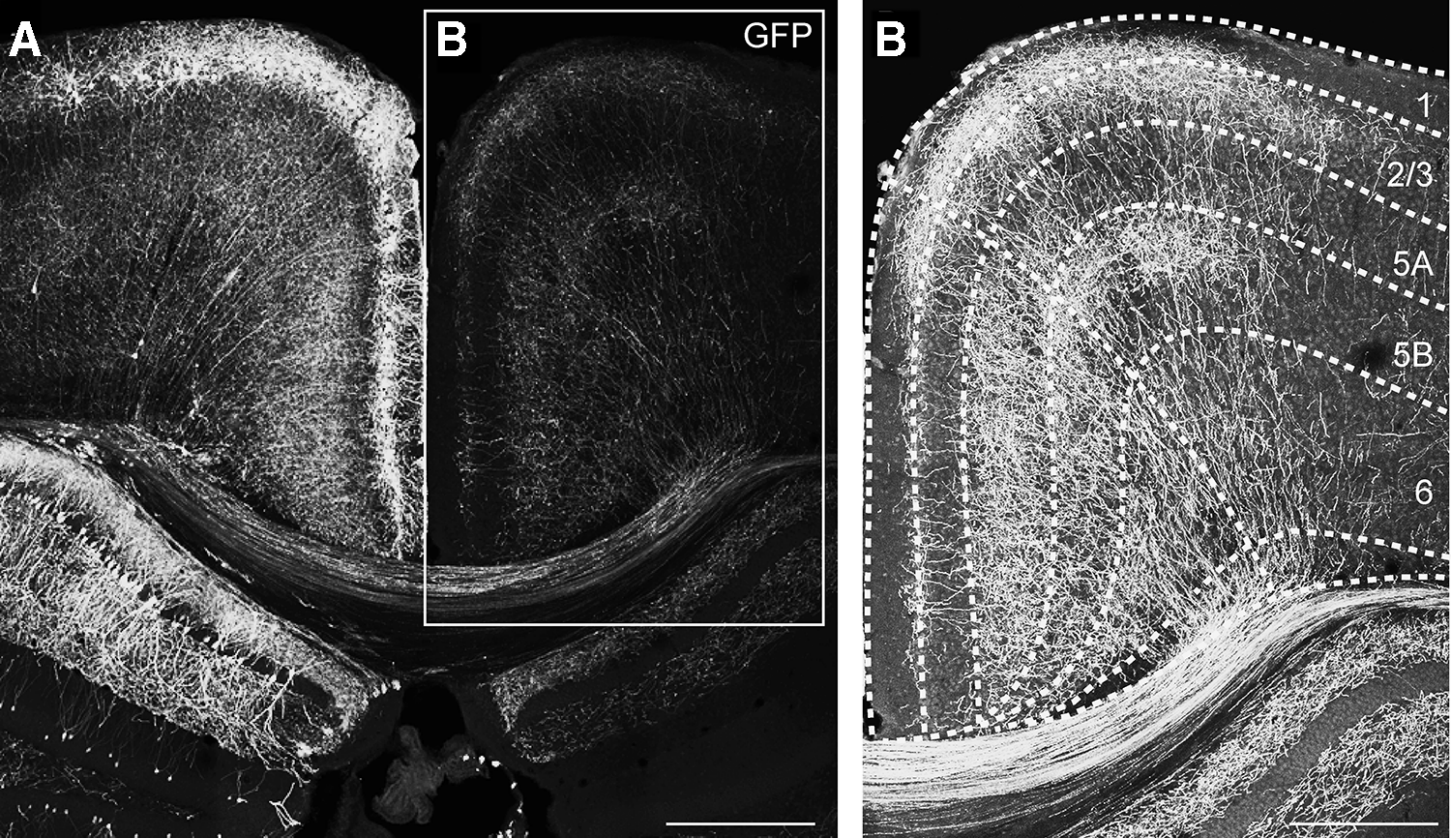
Branching of callosal axons in the retrosplenial cortex. Maximal projection of a confocal z-stack from a 40 µm slice of a P30 mouse brain electroporated with an eGFP-expressing plasmid at E16.5. Laminar boundaries were established according to DAPI counterstaining. Notice that in the neocortex, only neurons from superficial layers were electroporated (left panel), and that their axons cross the midline and invade the contralateral cortex (right panel). Scale bars 550 µm (panels in the left) and 350 µm (right panel)

To characterize the arborization of the terminal branches of callosal axons across the layers of the contralateral cortex, we performed unilateral in utero electroporation of E16.5 embryos with an eGFP-expressing plasmid (PCX-GFP). At this age, superficial pyramidal neurons are being produced in the ventricular zone of the developing neocortex (Mizuno et al. 2007; Wang et al. 2007). In postnatal coronal slices (P30), GFP+ neurons were found in the superficial layers of the RSC in the electroporated hemisphere (Fig. 6a). The axons of these neurons, which were also labeled, could be followed crossing the midline through the dorsal, but not the ventral part of the corpus callosum, as expected for a medial cortical region (Nishikimi et al. 2011). These axons invaded the contralateral homotopic cortex (Fig. 6b), where they developed a bimodal pattern of arborization. Similarly to what happens in other cortical areas (Yorke and Caviness 1975; Mizuno et al. 2007; Wang et al. 2007), extensive branching was observed in a territory including superficial layers and layer 5. Nonetheless, callosal terminal arbours specifically targeted the upper part of L5B, but not L5A and the lower part of L5B. This sublaminar-specific organization of callosal axons from superficial CPNs nicely fitted with the distribution of callosal PSP amplitudes observed in the different pyramidal neuron subtypes across layers, suggesting that our electrical stimulus on layers 2/3 was preferentially activating CPNs. In addition, the disposition of callosal terminal branches from superficial CPNs, likely overlapping with basal and apical dendrites of L2/3 and upper L5BL pyramidal neurons is likely to explain, at least in part, our former results.

### Larger feed-forward inhibitory currents recruited by callosal input in L2/3 and L5BL pyramidal neurons

**Fig. 7:**
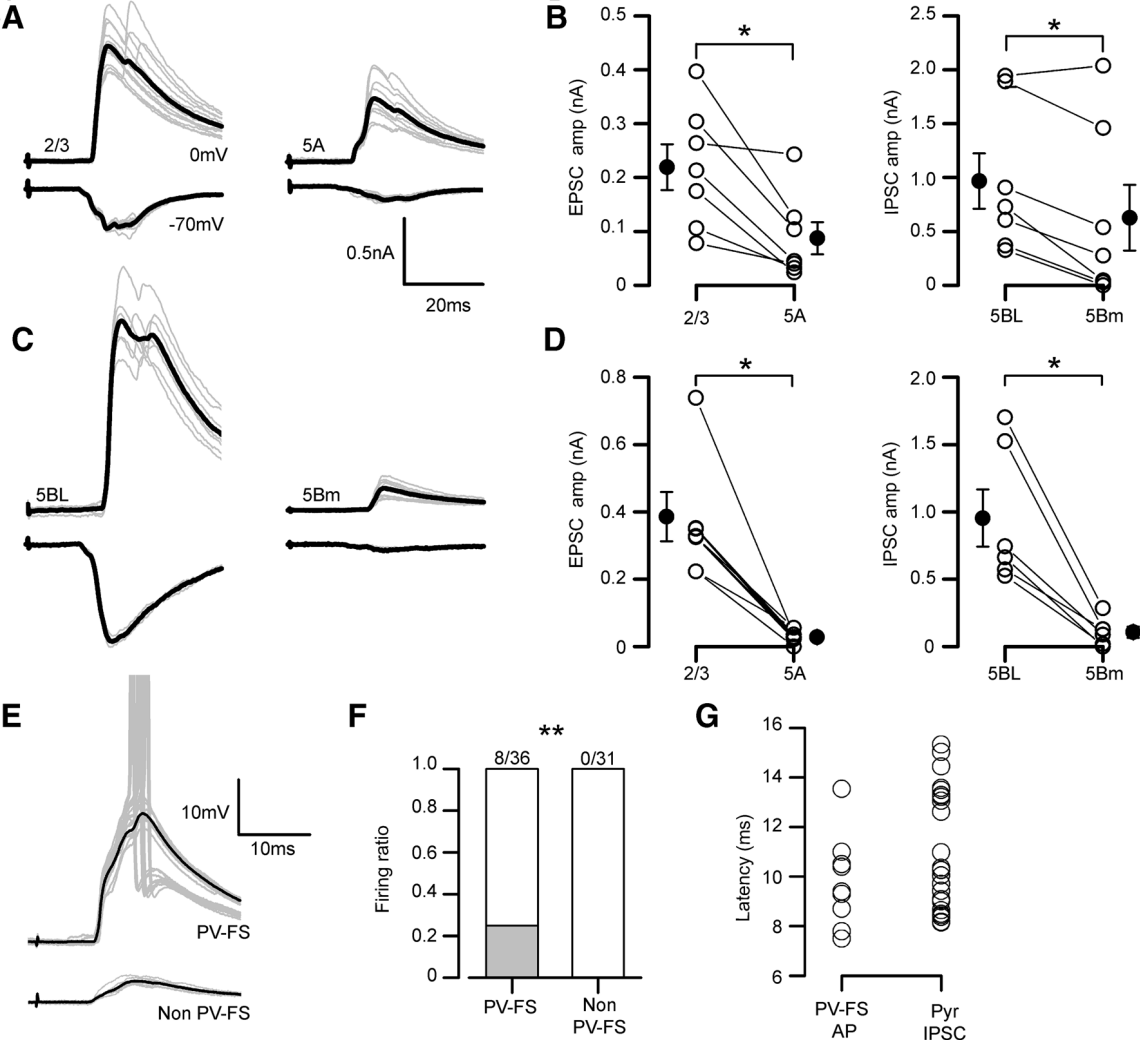
Inhibitory currents evoked on pyramidal neurons retain the specificity of callosal input and are dependent on PV-FS neurons. **a** EPSCs (lower traces in each panel) and IPSCs (upper traces in each panel) evoked in a L2/3 vs L5A pair sequentially recorded (stimulus intensity 200 µA). Gray traces are individual responses and the black trace is the average. **b** EPSCs (left panel) and IPSCs peak amplitudes (right panel) in a sample of sequential L2/3 vs 5Am pairs (*n* = 7, 6 slices from 5 mice). **c**, **d** Same as in panels **a**, **b** but for L5BL vs L5Bm sequential pairs (*n* = 6, 4 slices from 3 mice). All responses in **b** and **d** were evoked with 200 µA stimulation. **e** PSPs evoked in a PV-FS vs non PV-FS pair from layer 2/3 (stimulus intensity 200 µA). Grey traces are individual responses and the black trace is the average of the subthreshold responses. Notice that in some responses the PV-FS neuron reached the AP threshold (AP truncated). **f** Proportion of firing neurons in a sample of PV-FS and non PV-FS interneurons from the agranular RSC (only neurons from layers 2/3, 5A and 5B were included). **g** Comparison of the action potential latency (from stimulus artifact onset to action potential peak) of the sample of firing PV-FS neurons (*n* = 8) and the IPSC onset latency (from stimulus onset to 10% of IPSC peak amplitude) in the sample of pyramidal neurons from panels **b** and **d**. EPSCs were recorded at − 70 mV and IPSCs at 0 mV. In **a** and **c**, grey traces show the individual responses and the average is shown in black

In addition to targeting pyramidal cells, CPNs also synapse on contralateral inhibitory neurons (Carr and Sesack 1998; Cissé et al. 2003, 2007; Karayannis et al. 2007; Petreanu et al. 2007) which in turn innervate surrounding pyramidal cells, causing feed-forward inhibition. To characterize the organization of the feed-forward inhibition recruited by callosal input across the different pyramidal subtypes studied, we compared the IPSCs in sequentially recorded pairs of L2/3 vs L5A and L5BL vs L5Bm pyramidal neurons (Fig. 7a–d, *n* = 7 and 6, respectively). In these neuron pairs, the callosal EPSCs were also studied (EPSCs were recorded at − 70 mV and IPSCs at 0 mV, the measured reversal potential of the inhibitory and excitatory synaptic currents). IPSC peak amplitude was significantly smaller in L5A and L5Bm than in L2/3 and L5BL pyramidal neurons, respectively (Fig. 7a–d), mimicking the specific pattern of the callosal excitation on pyramidal neuron subtypes. This was particularly remarkable in L5BL–L5Bm pairs, which had their somas closely placed. In addition to the differences in the IPSCs, and consistently with our previous results, we observed that callosal EPSCs were larger on L2/3 and L5BL pyramidal cells with respect to L5A and L5Bm pyramidal neurons, respectively.

To determine which type of gabaergic interneurons were the source of this inhibitory input to pyramidal neurons, we studied the callosal responses in a sample of PV-FS and non PV-FS gabaergic neurons. Their distribution among layers was as follows: PV-FS neurons (*n* = 36): 10 in layer 2/3 (4 fired in response to contralateral EPSCs), 14 in layer 5A (3 fired in response to contralateral input), and 12 in layer 5B (1 fired in response to contralateral input). Non-PV-FS neurons (*n* = 31; none fired in response to contralateral input): 14 in layer 2/3, 8 in layer 5A, and 9 in layer 5B. We also recorded some PV-FS neurons in layer 6 (*n* = 8 PV-FS interneurons from 6 slices), but their responses to contralateral stimulation were lacking or very small (0.26 ± 0.29 mV; range 0–0.69 mV). Both cell types were distinguished according to their intrinsic electrophysiological properties (see “Materials and methods” and Tables 1, 2). Callosal PSPs evoked on non-PV-FS cells were smaller than in PV-FS interneurons (see example in Fig. 7e) and this difference resulted in that none of the recorded non-PV-FS interneurons fired in response to contralateral input (0/31); in contrast, as stated above, 8/36 PV-FS neurons, distributed in layers 2–5B, reached the action potential threshold in response to contralateral simulation (Fig. 7f). The latency of the action potentials fired by PV-FS interneurons (range 7.5–13.5 ms) overlapped but preceded the onset latency of the IPSCs evoked on pyramidal neurons (latency range 8.2–15.3 ms; Fig. 7g).

**Fig. 8:**
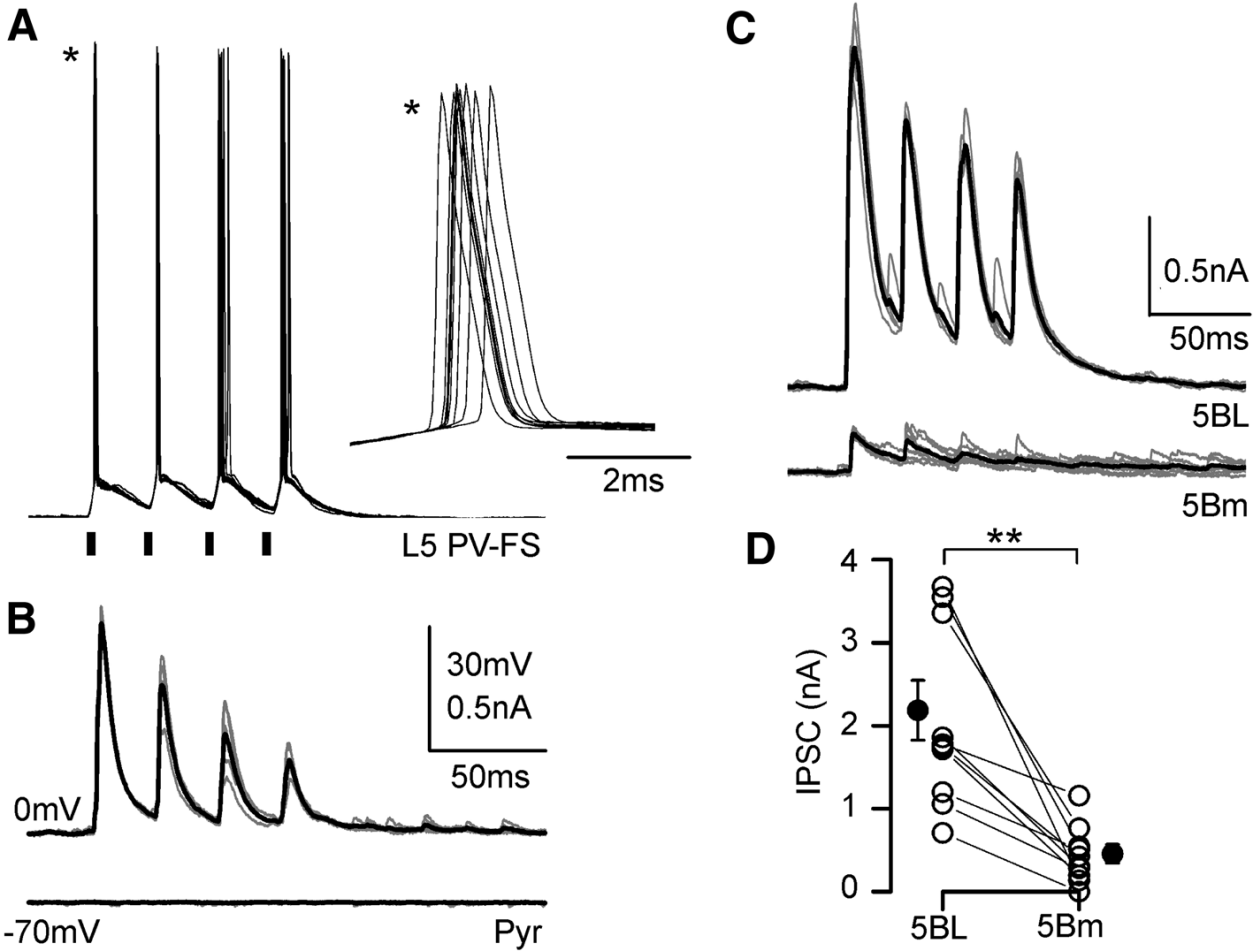
Channelrhodopsin-mediated recruitment of PV-FS interneurons triggers larger IPSCs in L5BL than in L5Bm pyramidal neurons. **a** Response of a PV-FS neuron (resting membrane potential − 70 mV) to a train of four consecutive flashes of 470 nm light (2 ms of duration separated by 25 ms; black bars under the recordings) applied through the ×40 objective; ten consecutive trials of four flashes superimposed. The inset (asterisks) shows superimposed, at a larger time scale, the first action potential of each trial. **b** IPSCs recorded in a pyramidal neuron (same slice than panel **a**) in response to trains of four consecutive flashes as those shown in **a**. The IPSCs are clearly seen at a holding potential of 0 mV but not at − 70 mV; five trials superimposed; each trial is shown in grey and their average is the blach trace. **c** IPSCs recorded at 0 mV in response to trains of flashes as in **a** recorded in a L5BL (upper traces) and a L5Bm neurons recorded simultaneously. The panel shows superimposed the responses to five consecutive trials; responses to individual trials shown in grey and their average in black. **d** peak amplitude of the IPSCs recorded in nine neurons pairs formed by a L5BL and a L5Bm neuron recorded sequentially. For each neurons the value shown is the average peak amplitude of the response to the first flash (*n* = 10 pairs; 4 slices from 3 mice of 23–25 postnatal days)

To reinforce our observation that the inhibition triggered by contralateral afferences was larger in L5BL than in L5Bm neurons we used Channelrhodopsin-2 photostimulation to specifically activate PV-FS neurons surrounding the recorded pyramidal neurons (Fig. 8). Pulses of blue light (470 nm; 2 ms of duration) were able to depolarize PV-FS neurons up to the threshold and to make them to fire action potentials (Fig. 8a); these action potentials evoked by photostimulation triggered IPSCs, but not EPSCs in pyramidal neurons (Fig. 8b). The IPSCs triggered by photostimulation were significantly larger in L5BL than in L5Bm pyramidal neurons (Fig. 8c, d; *n* = 9 neurons pairs sequentially recorded), in a way totally coincident with our result using electrical stimulation applied to the contralateral hemisphere (compare Fig. 7c, d with Fig. 8c, d). Due to the fact that channelrhodopsin-2 photostimulation activates, in a non-selective way, the PV neurons that are in the field of view of the 40× objective (and possibly axons from PV neurons whose soma is out of that area), these experiments show that the larger responses triggered by PV-FS interneurons in L5BL respect to L5Bm pyramidal neurons is not dependent on callosal input, but is a general property of the local circuits in layer 5 involving PV-FS interneurons. In some of the neuron pairs (4 out of 9) in which we tested the responses evoked by photostimulation we also recorded the synaptic responses evoked by contralateral electrical stimulation. The IPSCs evoked by electrical stimulation were also larger in L5BL than in L5Bm neurons (peak amplitude: 2006 ± 604 pA in 4 L5BL neurons and 69.3 ± 23.9 pA in 4 L5Bm neurons), which reinforces our previous results.

### Net inhibition on L2/3 pyramidal neurons

In our experiments, the stimuli applied to the contralateral cortex mostly evoked subthreshold PSPs in superficial pyramidal neurons. In response to stimuli of 200 μA, none of the 70 recorded L2/3 neurons fired action potentials (neurons recorded in 39 slices from 34 mice), a rate significantly lower than in upper L5BL neurons (4/14, *p* < 0.001).

**Fig. 9:**
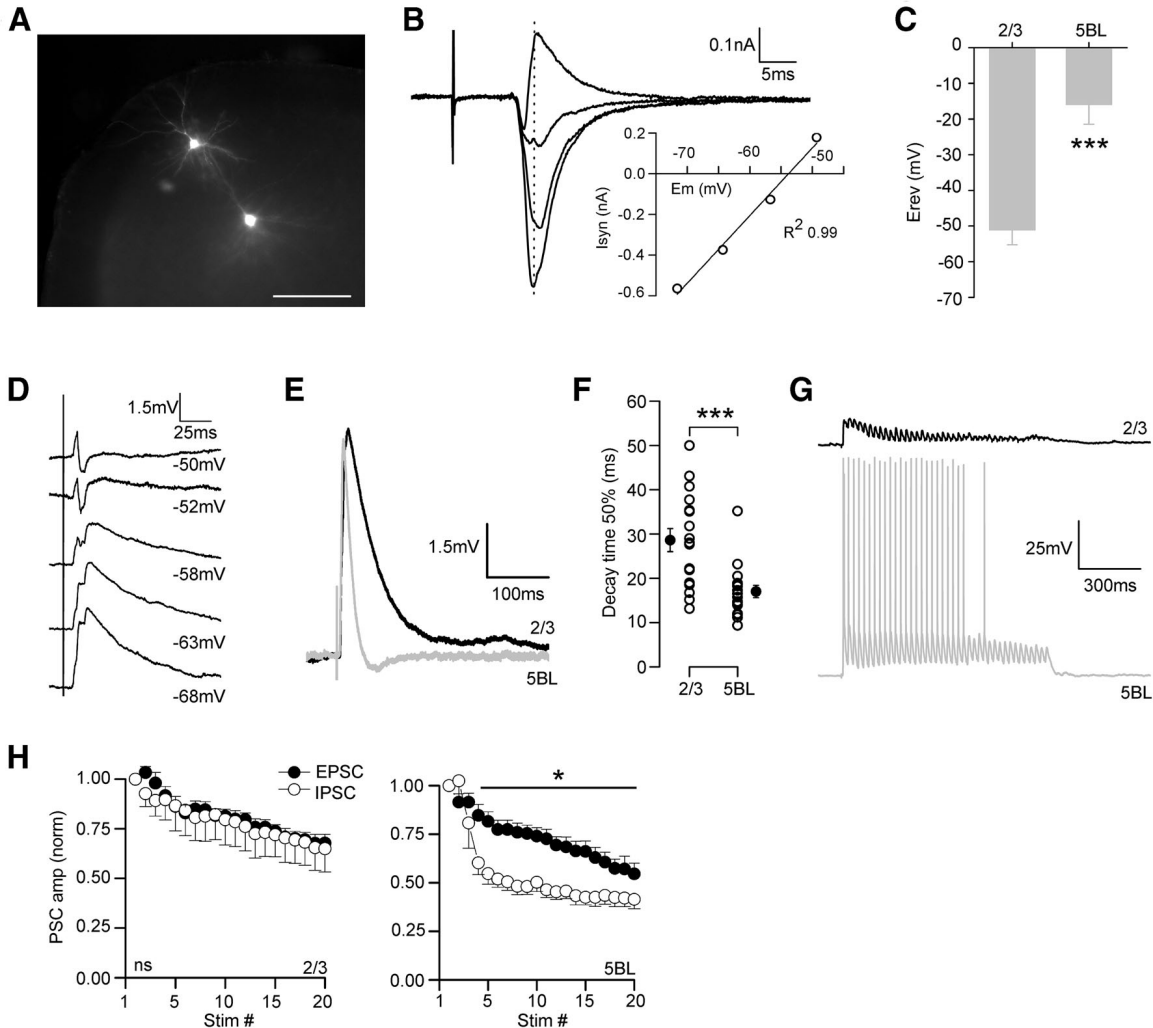
L2/3 pyramidal neurons are inhibited by callosal input. **a** Example of a pair of L2/3 vs L5BL pyramidal neurons sequentially recorded; scale bar 200 µm. **b** PSCs in the L2/3 pyramidal neuron shown in **a** in response to contralateral stimulation measured at different holding potentials. Each trace is the average of at least 5 consecutive responses. The inset shows the IV curve measured at the PSC peak using the responses recorded at the four most negative potentials. **c** Reversal potential of the callosal responses measured at the EPSC peak in a sample of L2/3 vs L5BL pairs (*n* = 6 pairs, 5 slices from 5 mice, stimulus intensity 200 µA). Note that the reversal potential of the callosal responses is more hyperpolarized on L2/3 pyramidal neurons. **d** Callosal PSP in a L2/3 neuron recorded at different membrane potentials. Notice that the response largely reverts at − 50 mV, below the action potential threshold. Each trace is the average of at least five consecutive responses. **e** Callosal PSPs in a L2/3 vs L5BL pair (same as shown in Fig. 3a). **f** Decay time to 50% of peak amplitude of the callosal PSP in a sample of L2/3 and L5BL neurons (same as shown in Fig. 3a–e; stimulus intensity 200 µA). **g** Callosal PSPs evoked in a sequential L2/3 vs L5BL pair in response to a 40 Hz train applied to the contralateral cortex (stimulus intensity 200 µA). **h** Short-term dynamics of the EPSCs and IPSCs evoked in a sample of L2/3 (upper) vs L5BL (lower) pairs (*n* = 8, five slices from five mice, stimulus intensity 200–500 µA)

This difference in the firing probability of L2/3 and upper L5BL neurons in response to contralateral stimulation could be caused by a lower excitatory/inhibitory balance of the callosal synaptic responses in L2/3 neurons. In fact, the reversal potential of the callosal response in L5BL neurons was clearly more positive than the threshold for firing, while in L2/3 was more negative (Fig. 9a–c, *n* = 6 L2/3 vs L5BL pairs sequentially recorded; see AP threshold values in Table 2) causing a net inhibition in these neurons (see example in Fig. 9d).

However, PSPs evoked on L2/3 pyramidal neurons had a longer decay time than those on L5BL neurons (Fig. 9e, f), predisposing them for stronger temporal summation. We argued then that in a context of sustained callosal activity, the recruitment of L2/3 pyramidal neurons could be supported by the temporal summation of successive inputs. To test this hypothesis, we applied trains of stimuli instead of single-pulse stimulation. In the range of 10–15 Hz stimulation, temporal summation of successive PSPs in a train was minimal in L2/3 pyramids (4th/1st PSP amplitude 1.10 ± 0.08, *n* = 15). Therefore, we used higher stimulation frequencies (trains of 20–40 pulses at 40 Hz) with the idea of shortening the interstimulus interval to facilitate the summation of successive responses. In 8 L2/3 vs L5BL pairs sequentially recorded, all L5BL neurons were recruited at some point during the train, while none of the L2/3 pyramidal neurons fired (see example in Fig. 9g). In a different paired sample of L2/3 vs L5BL pyramidal neurons, we repeated the experiment in voltage–clamp conditions. EPSCs and IPSCs depressed in both pyramidal neuron subtypes (Fig. 9h), but while in L2/3 pyramidal neurons EPSCs and IPSCs showed similar short-term dynamics (Fig. 9h left panel), in L5BL pyramidal cells, IPSCs depressed more than EPSCs (Fig. 9h right panel). This implied that the reversal potential of the response was maintained during the train in L2/3 pyramidal neurons, but became even more depolarized in L5BL pyramidal cells in response to sustained callosal input.

**Fig. 10:**
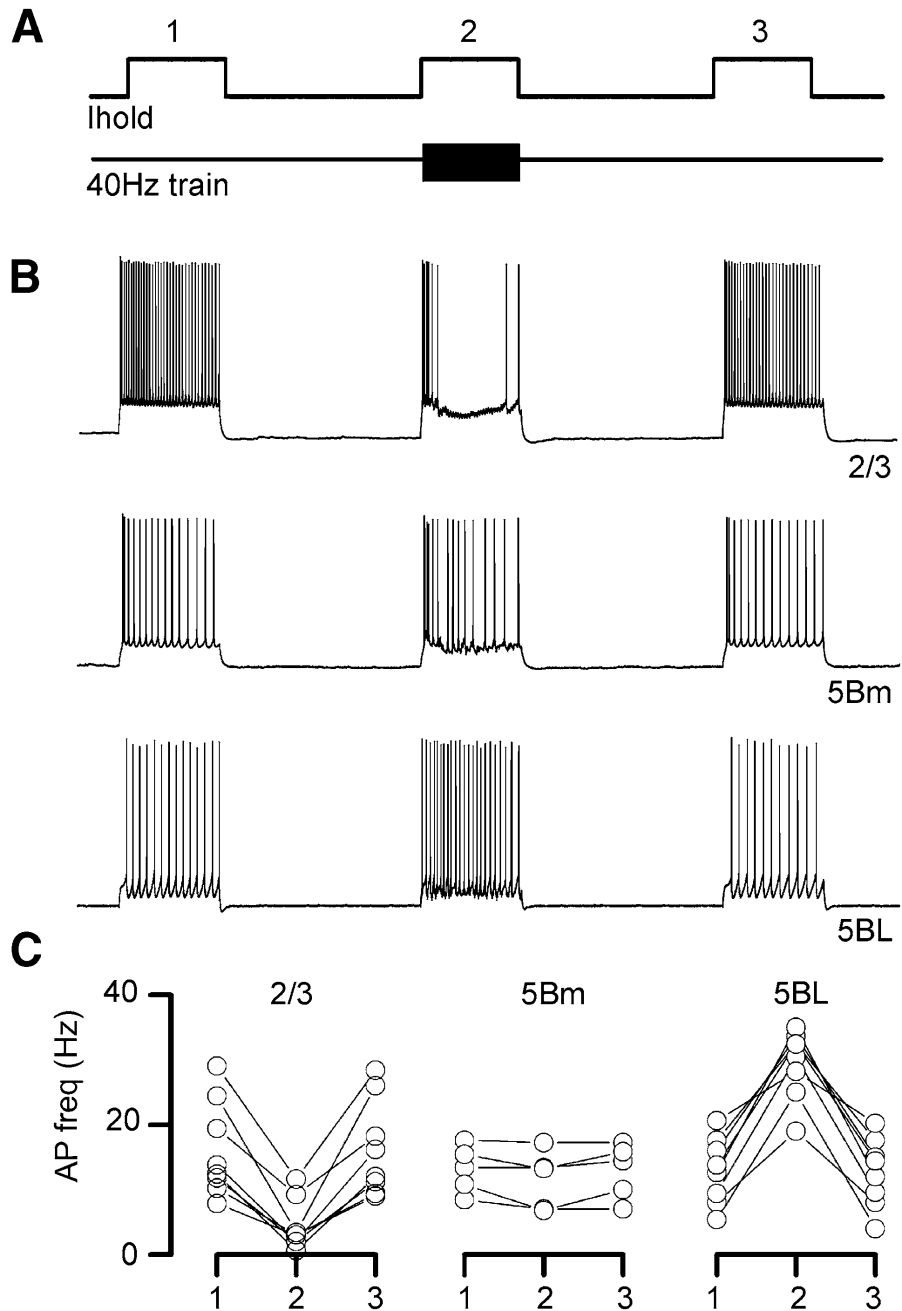
Different action of callosal input on layers 2/3 and 5 pyramidal neurons. **a** Experimental design: three suprathreshold current pulses (1 s duration) were injected in the recorded neuron with a 2 s interval; simultaneously to the second current pulse, a stimulus train was applied to the contralateral cortex (40 Hz, 1 s duration). **b** Representative examples of the effect of contralateral stimulation on the firing frequency in a L2/3 (upper panel), L5Bm (middle panel) and L5BL (lower panel). **c** Change in the firing frequency induced by 40 Hz stimulation of the contralateral cortex in a sample of L2/3 (*n* = 8), L5Bm (*n* = 5) and L5BL (*n* = 8) pyramidal neurons. Stimulus intensity 200 µA

Even more, in a different sample of non-paired recordings, we tested the effect of 40 Hz train of contralateral stimuli on the firing activity evoked in the recorded neuron by the injection of a suprathreshold current pulse (Fig. 10). In all L2/3 pyramids tested (*n* = 8), the firing rate was decreased by contralateral stimulation. The opposite pattern was observed in L5BL neurons (*n* = 8), and, as expected from their low responsiveness to callosal input, no change in the number of APs was detected in L5Bm pyramidal neurons (*n* = 5). Altogether, these data indicates that, in our conditions, the firing activity of CPNs in one hemisphere suppresses the activity of contralateral superficial pyramidal neurons.

## Discussion

### Origin of the synaptic responses studied

We have employed an extracellular electrical stimulation approach to study callosal synaptic responses. A potential problem with extracellular stimulation is the antidromic activation of neurons projecting to the recording site, which is exacerbated in our case given the reciprocal nature of callosal connections. However, in our experimental conditions the contribution of responses caused by the antidromic stimulation of CPN was minimal when using stimulus intensities of 200 μA. The proportion of recorded neurons showing antidromic spikes quantified in a large sample of neurons was very low: in superficial neurons, which include most CPN (Fame et al. 2011), less than 4% responded antidromically to stimuli of 100 and 200 μA and in layers 5 and 6 0% responded antidromically. In those neurons in which we detected antidromic spikes in response to contralateral cortex stimulation, synaptic responses were elicited with stimuli of lower intensity than that required to evoke an antidromic spike (see Fig. 1e). All data relevant to support our conclusions (Figs. 4 panels e–h, 5, 7, 9 panels b–g, 10) were obtained with stimulus intensities of 200 μA. In these conditions, postsynaptic responses (PSPs, EPSCs and IPSCs) showed sizeable differences among different types of pyramidal neurons, suggesting that they were caused by callosal input and not by ipsilateral neurons recruited antidromically. For instance, note in Fig. 4c that the bimodal shape of the callosal PSP amplitude was already present in PSPs evoked by the lower stimulus intensity used (100 μA). These points show that the overall contribution of synaptic responses caused by antidromic stimulation was minimal.

A second major consideration was the laminar origin of the callosal input being studied. As already mentioned, it is known that most CPNs are located in superficial layers (Fame et al. 2011), and our stimulus electrode was directly placed on these neurons, suggesting a strong bias for this source with respect to the minor populations of CPNs in layers 5 and 6. In addition, we studied the arborization of callosal axons originated in superficial CPNs of the agranular RSC. Their terminal branches occupied two strips, in the boundary of layers 1 and 2 and in the upper part of layer 5B (but not in the lower 5B; see Fig. 5b). This distribution nicely fitted with the specificity of the responses recorded in response to contralateral stimulation across pyramidal neurons, with those in layers 2/3 and upper layer 5B showing the larger responses. All together, this strongly pointed to the fact that the observations reported here reflect the properties of the callosal input originated in superficial layers.

### Columnar distribution of callosal response amplitude in contralateral pyramidal neurons

Our results clearly show that in the agranular RSC, CPNs had a larger impact in contralateral L2/3 and L5BL pyramidal neurons. Postsynaptic responses (PSPs, EPSCs and IPSCs) elicited by CPNs were larger on these neurons than in other pyramidal subtypes, including L5A, L5Bm and L6 ones. In addition, and as suggested by the branching of callosal axons from superficial CPNs on the upper, but not lower part of layer 5B, we have shown that responses on upper L5BL pyramidal cells are larger than on those laying in the lower half of layer 5B. This result points to the existence of a functional segregation within this population. Similarly, in local circuits of the motor cortex, superficial pyramidal neurons trigger larger responses in those L5B corticospinal pyramidal neurons located closer to the boundary with L5A (Anderson et al. 2010).

Our data are not conclusive regarding the mechanisms underlying the observed distribution of the size of the callosal responses among different types of pyramidal neurons; however, the observation that callosal inputs had a stronger impact in L5BL than in L5Bm pyramidal neurons may be explained by a preferential connectivity of callosal axons with the L5BL neurons. This hypothesis is supported by the recent discovery of a specific molecular recognition mechanism controlling the interaction between axons from L2/3 pyramidal cells and postsynaptic compartments in the thick-tufted pyramidal neurons of layer 5 (Harwell et al. 2012). This interaction is not exclusive of local circuits but also applies to callosal axons from contralateral superficial CPNs (Harwell et al. 2012). This is also in line with a study showing that in its home cortical column, superficial pyramidal neurons have a tenfold larger connection probability with the large bursting pyramidal neurons than with layer 5 regular-spiking medium-size pyramidal neurons (Thomson and Bannister 1998). Nonetheless, our results could be explained by other mechanisms that do not require a selectivity of the innervation of contralateral targets by callosal axons. Both L5BL and L5Bm pyramidal neurons have basal and apical dendrites in the target region of callosal axons (layer 1/2 boundary and upper layer 5B), but L5BL pyramidal neurons have larger dendritic arbors than L5Bm neurons. If callosal connectivity were based on a probabilistic function of axodendritic overlap, then larger EPSCs should be also expected in thick-tufted with respect to thin-tufted neurons.

### Influence of callosal input in local cortical inhibitory networks

In agreement with previous evidence (Carr and Sesack 1998; Cissé et al. 2003, 2007; Karayannis et al. 2007; Petreanu et al. 2007), we show that PV-FS and non PV-FS gabaergic interneurons from layers 2/3 and 5 received direct callosal input and that IPSCs were evoked on pyramidal neurons in response to contralateral stimulation. In addition, we show that among gabaergic interneurons and in response to single-pulse stimulation, spikes were evoked only in PV-FS cells. Even more, the AP latency in the recruited PV-FS cells largely overlapped but preceded the IPSC onset in pyramidal cells. The absence of evoked spikes in our recorded sample of non PV-FS interneurons, and the matching of the latency of the spikes triggered in the PV-FS sample with the onset latency of the IPSCs recorded in pyramidal cells strongly suggests that these interneurons were the main source of the inhibition evoked on pyramidal cells.

Our results are in agreement with a study demonstrating that in the prefrontal cortex, layer 5 PV-FS interneurons driven by callosal inputs preferentially target thick-tufted but not thin-tufted pyramidal neurons in layer 5 (Lee et al. 2014). However, this preferential innervation of thick-tufted neurons is not coincident with other report studying the contribution of callosal input in layer 5 circuits of the auditory cortex (Rock and Apicella 2015); these authors show that a larger PV-FS dependent inhibitory input was triggered by callosal input on layer 5 corticocortical (medium-size regular spiking pyramidal neurons) vs corticocollicular pyramidal neurons (bursting cells with large somas). This divergence among studies, including our results, suggests that the local structure of the callosal circuits is highly specialized across different cortical areas, particularly in layer 5. In our experiments, photostimulation of PV-FS neurons resulted in larger IPSCs in L5BL with respect to L5Bm pyramidal neurons, indicating that stronger PV-FS dependent feed-forward inhibition in the former was not a particular feature of the callosal projection, but a general property of the organization of retrosplenial local microcircuits.

### A laminar-dependent effect of callosal input

We have demonstrated that, in our conditions, the reversal potential of the callosal response in L2/3 pyramidal neurons is more negative than the AP threshold, making unlikely their recruitment in response to this type of stimulus. Notice that in our recordings, the Nernst equilibrium potential for Cl^−^ was about − 70 mV, while in physiological conditions, it may be closer to − 85 mV, and therefore, we expect the physiological reversal potential of the callosal response to be still more hyperpolarized. Importantly, the exact same situation described here also applies for the response of superficial pyramidal neurons to local input arising from groups of neighbor superficial cells (Mateo et al. 2011; Avermann et al. 2012).

We also failed to detect an increase in the recruitment of superficial pyramidal cells in response to 10–15 and 40 Hz trains of callosal input. Moreover, the firing activity evoked in these neurons by intracellular current injection was reduced when a train of contralateral input was simultaneously applied, demonstrating a direct inhibitory effect of the callosal input on these neurons even in a context of sustained presynaptic activity, similarly to what occurs during an up state. Indeed, under 40 Hz stimulation, EPSCs and IPSCs similarly depressed on L2/3 pyramidal cells, suggesting that the reversal potential of the response was maintained even in these conditions.

In contrast, L5BL pyramidal neurons in upper layer 5B often responded with suprathreshold PSPs to contralateral input. The dense code implemented by these neurons partially depends in their special intrinsic electrophysiological properties (Lee et al. 2014), but we also show that the reversal potential of the callosal response in these neurons was more positive than their threshold potential for firing, suggesting a role for synaptic mechanisms. In the barrel cortex, the excitatory to inhibitory balance of the synaptic currents elicited by local input is more favorable to excitation on pyramidal neurons in layer 5 vs those of layers 2/3 (Adesnik and Scanziani 2010), most likely leading to a more depolarized reversal potential of the synaptic response in the formers, as in our case. The difference observed in the response properties among L2/3 and L5BL pyramidal neurons were further increased in response to 40 Hz trains of contralateral stimuli, as a result of the stronger depression of IPSCs on the latter. In the future, it would be of interest to investigate the mechanisms underlying this observation. Overall, the tight similarities between the properties of the responses of L2/3 and L5BL pyramidal neurons to contralateral CPNs described here and the properties of the responses of these neurons to local inputs from superficial pyramidal neurons of the same cortical column reported in other studies further support the hypothesis of an integrative role of the callosal projection in the retrosplenial cortex.

## Electronic supplementary material

Below is the link to the electronic supplementary material.

Supplementary material 1 (PDF 109 kb)
