# Supplementary material for: Callosal responses in a retrosplenial column
Source: Brain Struct Funct. 2017 Oct 28;223(3):1051–69. doi: 10.1007/s00429-017-1529-5 (PMC5869903; doi:10.1007/s00429-017-1529-5)
Supplement: Supplementary file 1 — Supplementary material 1 (PDF 109 kb) [file 429_2017_1529_MOESM1_ESM.pdf]

Online Resource

Brain Structure and Function

Callosal responses in a retrosplenial column

Alejandro Sempere-Ferràndez, Belén Andrés-Bayón and Emilio Geijo-Barrientos

Corresponding author:

Emilio Geijo-Barrientos

Instituto de Neurociencias. Universidad Miguel Hernández – Consejo Superior de Investigaciones Científicas. Campus de San Juan. Avenida Ramón y Cajal s/n, San Juan de Alicante. 03550 Alicante, Spain

Tel: +34 96 591 9534

Fax: +34 96 591 9560

Email: emilio.geijo@umh.es

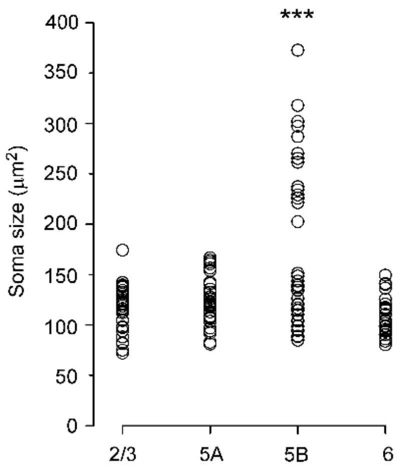

Online Resource Figure 1. Somatic size of neurons in layers 2/3, 5A, 5B and 6. We measured the area of the soma from 40x DIC microphotographs obtained during the recording sessions (see examples in Fig. 2C, main text). We compared the somatic size of the neurons in the four layers with an ANOVA analysis, which revealed significant differences across groups (F=11.45; p < 0.001). A Bonferroni test revealed significant differences in the somatic size of layer 5B cells with respect of those in layers 2/3, 5A and 6 (***p<0.001 for all comparisons). No statistically significant differences were detected among the other three layers. This analysis shows that the average somatic area of neurons in layer 5B was different from the area of the neurons in the other layers; the somatic area mean values are given in Online Resource Table 1 below. Note that while the area of the somas measured in layers 2-3, 5A and 6 are quite similar, and always below 200µm^2, in layer 5B there is a group of cells with larger somas (> 200µm^2). The presence of neurons with larger somas in layer 5B made us consider two categories within this layer: layer 5B large (L5BL) and layer 5B medium-size (L5Bm).

Online Resource Table 1. Somatic area of neurons in the agranular cortex of mice.

Somatic area of neurons in the agranular cortex of mice			
**Layer**	**n**	**mean±s.d. (µm^2)**	**Range (µm^2)**
2/3	32	117.7 ± 23.1	72.1 - 174.2
5A	30	124.3 ± 24.1	81.0 - 166.6
5B	35	176.6 ± 81.0	84.8 – 372.8
6	27	109.7 ± 18.1	80.3 - 149.5

The table shows the average values and the range of the somatic area of all neurons included in Online Resource Fig. 1 calculated by layers. Data given as mean ± s.d.

Online Resource Table 2. Intrinsic properties of L5BL and L5Bm pyramidal neurons.

Intrinsic properties of L5BL and L5Bm pyramidal neurons													
	**n**	**Vrest (mV)**	**Rm (MΩ)**	**Vsag**	**Tau (ms)**	**Rehobase (pA)**	**APth (mV)**	**Vrest-APth (mV)**	**AP amp (mV)**	**AP half-width (ms)**	**Burst ratio (%)**	**Burst freq (Hz)**	**No burst freq (Hz)**
**L5BL**	51	-64.8 ±2.6	43.9 ±12.3	0.26 ±0.04	12.9 ±0.3	222.7 ±113.1	-42.7 ±3.0	-17.7 ±13.9	96.2 ±7.3	0.53 ±0.09	45	277.3 ±57.8	9.1 ±14.5
**L5Bm**	15	-64.6 ±4.1	198.9 ±53.2	0.16 ±0.04	26.5 ±7.4	68.0 ±25.7	-40.8 ±4.0	-23.8 ±7.0	91.9 ±3.9	0.66 ±0.07	0	--	17.8 ±15.4
		ns	***	***	***	***	ns	ns	**	***	**		

Electrophysiological properties of all pyramidal neurons recorded in layer 5B. Most parameters were measured as described in tables 1 and 2 of the main text. Rheobase was measured as the size of the smallest depolarizing current pulse able to drive the cell to the action potential threshold. To determine the presence of burst of action potentials we measured the instant frequency of the two first action potentials fired by a just supra-threshold current pulse; a response was considered a burst when the two initial spikes had an instant frequency >150 Hz as shown in figures 2d-e and 3d of the main text. With this criterion we separated burst firing neurons (Burst freq; L5BL range 179.2 – 377.4 Hz) and neurons that did not fire bursts (No burst freq; L5BL range: 9.1 -76.5 Hz; L5Bm range: 4.9 – 58.8 Hz). Data given as mean ± s.d. Statistical comparisons described in the methods section of the main text.
